# Plant growth Enhancement in Colchicine-Treated Tomato Seeds without Polyploidy Induction

**DOI:** 10.1007/s11103-024-01521-1

**Published:** 2024-12-12

**Authors:** Rosa Irma Obando-González, Luis Enrique Martínez-Hernández, Leandro Alberto Núñez-Muñoz, Berenice Calderón-Pérez, Roberto Ruiz-Medrano, José Abrahán Ramírez-Pool, Beatriz Xoconostle-Cázares

**Affiliations:** Departamento de Biotecnología y Bioingeniería, Centro de Investigación y de Estudios Avanzados del Instituto Politécnico Nacional, Ciudad de Mexico, 07360 México

**Keywords:** Genetic diversity, Transcriptome, Single nucleotide polymorphisms, Plant hormones, Plant vigor

## Abstract

Plant breeding plays a pivotal role in the development of improved tomato cultivars, addressing various challenges faced by this crop worldwide. Tomato crop yield is affected by biotic and abiotic stress, including diverse pathogens and pests, extreme temperatures, drought, and soil salinity, thus affecting fruit quality, and overall crop productivity. Through strategic plant breeding approaches, it is possible to increase the genetic diversity of tomato cultivars, leading to the development of varieties with increased resistance to prevalent diseases and pests, improved tolerance to environmental stress, and enhanced adaptability to changing agroclimatic conditions. The induction of genetic variability using antimitotic agents, such as colchicine, has been widely employed in plant breeding precisely to this end. In this study, we analyzed the transcriptome of colchicine-treated tomato plants exhibiting larger size, characterized by larger leaves, while seedlings of the T2 generation harbored three cotyledons. A total of 382 differentially expressed genes encoding proteins associated with anatomical structure development, hormone synthesis and transport, flavonoid biosynthesis, and responses to various stimuli, stresses, and defense mechanisms were identified. Gene enrichment analysis suggests a role for auxin and flavonoid biosynthesis in cotyledon formation. Furthermore, single-nucleotide polymorphisms were mapped in colchicine-treated plants and determined which corresponded to differentially- expressed genes. Interestingly, most were associated to only a few genes in a similar location. This study provides significant insights into the genes and metabolic pathways affected in colchicine-treated tomatoes that exhibit improved agronomic traits, such as plant vigor and improved photosynthesis rate.

**Supplementary Information:**

The online version contains supplementary material available at 10.1007/s11103-024-01521-1.

## Key message

Commercial crops, including tomato cultivars, are negatively impacted by biotic and abiotic stresses. Increasing genetic diversity through plant breeding can develop tomato varieties with enhanced disease and pest resistance, better tolerance to environmental stress, and adaptability to changing agroclimatic conditions. The present study employed the antimitotic agent colchicine to induce genetic variability. Three-cotyledon seedlings in the T2 generation developed adult plants displaying a vigorous phenotype with larger leaves. Transcriptome analyses identified 382 differentially expressed genes associated with anatomical structure development, hormone synthesis and transport, flavonoid biosynthesis, and responses to various stimuli, stresses, and defense mechanisms. The analysis suggests that auxin and flavonoid biosynthesis could play a role in cotyledon formation. The identification of single-nucleotide polymorphisms in colchicine-treated plants demonstrated the capacity to increase genetic diversity and reshape gene expression without altering genome content. The study provides insights into genes and metabolic pathways modulated in colchicine-treated tomatoes, leading to improved agronomic traits such as plant vigor and enhanced photosynthesis rates.

**Supplementary Information:**

The online version contains supplementary material available at 10.1007/s11103-024-01521-1.

## Introduction

Tomato (*Solanum lycopersicum* L.) is a dicotyledonous plant native to Central and South America, consumed worldwide. Tomato breeding is a critical aspect of agricultural research, particularly for addressing the abiotic and biotic stress challenges faced by this globally significant crop (Singh et al. 2017). Abiotic stress, such as extreme temperatures, drought, and soil salinity can severely affect tomato crop productivity, leading to reduced yields and compromised fruit quality. To address these problems, breeders have focused on developing tomato varieties with enhanced tolerance to these environmental challenges, incorporating traits from native cultivars to cope with elevated temperatures and drought, and to improve nutrient uptake. Additionally, the threat of pests and diseases poses a constant challenge. Tomato is susceptible to over 200 diseases and insects that can devastate entire crops, including emergent and recently evolved pathogens (Ma et al. 2023). Breeding efforts aim to create resistant varieties by incorporating genes that confer immunity or tolerance to common pests and diseases, which could reduce disease management with chemical pesticides (Van Esse et al. 2020). The ongoing development of resilient and high-yielding tomato cultivars is crucial for ensuring global food security and sustainable agricultural practices in a constantly changing environment. Through strategic breeding programs, it is desirable to increase genetic variability within the tomato germplasm pool, facilitating the identification and incorporation of key genes and alleles conferring tolerance or resistance to specific stress factors (Foolad 2004).

Tools for increasing genetic variability include the induction of polyploidy, a common phenomenon in plants which refers to having multiple sets of chromosomes (Zhang et al. 2019; Nieto Feliner et al. 2020). Polyploidy can arise through several mechanisms, including genome duplication and interspecific hybridization. The prevalence of polyploidy in plants has been reported to contribute to their adaptability and evolutionary success because it can result in an increase of genetic diversity and the emergence of new traits (Zhang et al. 2019). Furthermore, polyploidy has been associated with various agronomic benefits, including increased yield and disease resistance (Azzi et al. 2015; Van De Peer et al. 2021). Therefore, there are many chemicals used for the induction of polyploidy like colchicine (Zhou et al. 2017). This antimitotic agent has a direct effect on gene copy number and chromosome sets and can affect gene expression, biomass, cell size, and organ size, among others (Zhu et al. 2021; Zhang et al. 2019; Zhao et al. 2017; Chen et al. 2021). Genetic alterations induced by colchicine treatment involve chromosomal rearrangements, single nucleotide polymorphisms (SNPs), amplification of repetitive sequences, or loss of sequences, although the molecular mechanisms underlying these changes remain unknown (Del Pozo & Ramirez-Parra 2015).

In recent years, advances in sequencing technologies have allowed for more comprehensive analyses of polyploid genomes, providing insights into the mechanisms of polyploidy in plants (Chen et al. 2020). High-throughput sequencing technologies have enabled the identification of novel polyploid-specific genes and regulatory elements (Aversano et al. 2012). Transcriptome analysis has also facilitated the identification of candidate genes underlying polyploid-associated traits, such as increased biomass, stress tolerance, and disease resistance (Aversano et al. 2012; Tossi et al. 2022). The integration of transcriptome analysis with other genomic and phenotypic data would provide a more comprehensive understanding of the mechanisms and consequences of polyploidy in plants.

In the present study, we employ tomato plants to better understand the mechanisms underlying genetic modifications induced by colchicine treatment, which could explain at molecular level desirable genetic variations and could also lead to the development of new phenotypes desirable for agriculture.

## Materials and methods

### Plant material and colchicine treatment

Tomato seeds (*Solanum lycopersicum* var. Rio Grande) were treated with 0.1% colchicine solution for 48 h followed by rinsing with water three times 10 min each wash and germinated in seedbeds. Flow cytometry was used to confirm the ploidy level in T0, T1 and T2 generations, while the phenotype of the plants was monitored for the three generations (T3). Tomato plants were grown in a greenhouse using a mixture of peat, soil, and perlite (24:24:1), irrigated to field capacity at a temperature ranging from 18–32 °C and a light intensity of 850 PAR (photosynthetically active radiation).

## Nuclei extraction and flow cytometry analysis

Foliar samples from tomato plants after one month of development, along with the untreated control plants and soybean (*Glycine max*), employed as a standard for DNA content calculation. Sections of 0.5 to 1 cm^2^ were taken from leaves and mechanically disintegrated for 1 min using a scalpel and 400 µl of Sysmex CyStain UV Precise P extraction buffer. Subsequently, 2 µl of RNase A (10 mg/ml) and 10 µl of dithiothreitol (DTT, 1 M) were added. The solution was transferred to a tube with a disposable 30 μm nylon mesh filter, and 900 µl of 1X PBS, 2 µl of RNase A (10 mg/ml), and 10 µl of DTT (1 M) were added. Finally, 100 µl of propidium iodide (PI, 0.5 mg/ml) was added, and the mixture was incubated for 20 min at room temperature in darkness before flow cytometry analysis. Genome size was determined using the LSR Fortessa flow cytometer (Dickinson, Franklin Lakes, NJ). PI fluorescence was generated by excitation with a 50-mW argon laser (Saphire, Coherent, Santa Clara, CA) and emission collection at 490 nm with a 585 nm band-pass filter. Kaluza Analysis 2.1 software (Beckman Coulter, Indianapolis, IN) was used for cytogram analysis. Nuclei corresponding to the area of interest were framed, excluding signals emitted by cellular debris. The genome size ratio was calculated using the equation reported previously (Dolezel & Bartos 2005).

## Total chlorophyll content

Chlorophyll extraction was based on the protocol described by (Arnon 1949) with some modifications. 1 cm^2^ leaf tissue sections from three different positions (lower, middle, and upper) of the analyzed plants were taken and weighted. 200 µl of phosphate buffer (25 mM KH_2_PO_4_, *pH* 7, with the addition of 2 mM EDTA, pH 8, after pH adjustment) was added, followed by homogenization with a pestle, and 800 µl of 80% acetone was added. It was incubated for 1 h at room temperature with constant shaking, then centrifuged for 30 min at 13,000 rpm at room temperature. Finally, the absorbance of the supernatant was measured at 645 and 663 nm, using 200 µl of phosphate buffer and 800 µl of 80% acetone as a blank. Statistical analyses were calculated using Student’s *t*-tests for parametric samples.

## Photosynthetic rate measurement

Photosynthetic rate measurements were conducted using the portable photosynthesis system LI-6400XT by LI-COR on six different leaves from each plant to be analyzed, following a schedule between 9:00 and 11:00 am and maintaining a consistent light intensity. Light and temperature were measured in the greenhouse where tomato plants were grown, and those parameters were employed to set similar conditions in the portable photosynthesis apparatus for estimating the plant photosynthetic rate. The light intensity value was generally adjusted to 800 µmol/s, and the desiccant was placed in the middle range. CO_2_ control was based on a reference of 400 µmol/mol, the coolers were turned on, and a temperature of 20 to 25 °C was established. Each leaf was inserted into the IRGA chamber, ensuring no leaks, and setting an area of 6 cm^2^ with an average stomatal conductance (stomatal radius = 1). Flow control was monitored during measurements, maintaining constant humidity.

## RNA extraction

RNA extraction from leaf tissue was performed using the Direct-zol™ RNA Miniprep Plus Kit (Zymo Research) following the manufacturer’s specifications. Quantification and the 260/280 absorbance ratio were determined using the Nanodrop One spectrophotometer (Thermo Scientific, Waltham, MA, USA). The integrity of the extracted RNA was assessed on a 1.4% denaturing agarose gel, and visualization was performed using the BioDocAnalyzer gel documentation system (Biometra GmbH, Jena, Germany).

## Massive RNA sequencing (RNA-Seq)

Leaf tissue from two third-generation plants with the tricotyledonous phenotype treated with colchicine and control plant were used. The RNA was treated with DNase I according to the recommendations of the manufacturer (Invitrogen, Carlsbad, CA, USA). The samples were then sent to Macrogen, Inc., Korea, for massive sequencing. Sequencing libraries were generated using the Illumina platform (Illumina HiSeq2500), obtaining 20 million paired-end mRNA reads in each sample.

## Bioinformatic analysis

The quality of the reads and absence of adapters were assessed using the FastQC program (https://www.bioinformatics.babraham.ac.uk/projects/fastqc/, accessed on July 23, 2022). Data processing was performed using the Linux operating system and R packages. The reads were mapped to an assembled tomato transcript (GCF_000188115.5) obtained from the NCBI genomic database using the RSEM package. This program performed transcript assembly and abundance estimation, producing a normalized profile in TPM (transcripts per million) and FPKM (fragments per kilobase of transcript per million mapped reads). The resulting counts were processed to obtain differential expression using the R/Edge R package, estimating genetic dispersions by conditional maximum likelihood and an empirical Bayes model to shrink the dispersions towards a consensus value. Finally, differential expression was evaluated for each transcript using an exact test analogous to Fisher’s exact test (Robinson et al. 2010). A false discovery rate (FDR) of 0.05 and an absolute fold-change ≥ 2 were used. A heat map was constructed to compare DEGs between the conditions. The web server GetOrf (https://www.bioinformatics.nl/cgi-bin/emboss/getorf, accessed on August 2, 2022) was used to identify and extract possible open reading frames (ORFs) and their translation to amino acid sequences. For subsequent analyses, the acquired sequences underwent functional annotation by attributing them the function that best matched the results of the BLASTp alignment (https://blast.ncbi.nlm.nih.gov/Blast.cgi?PAGE=Proteins, accessed on August 15, 2022) by applying an E-value threshold of 10^–5^. *Arabidopsis thaliana* reference sequence served as the template genome for the analysis.

## Gene enrichment analysis

The enrichment analysis was carried out using the Plant Gene Set Enrichment Analysis Toolkit (PlantGSEA) web server (http://systemsbiology.cau.edu.cn/PlantGSEA/, accessed on August 17, 2022). Gene assignment was performed in the homologous species *A. thaliana* and genes were identified for the control, dicotyledonous tomato plants and tricotyledonous T2 tomato plants. Each gene list was analyzed separately using the same server. Gene ontology analysis was conducted using the Gene Ontology Resource (http://geneontology.org/, accessed on August 10, 2022) and the Tair GO annotation search web server (https://www.arabidopsis.org/tools/bulk/go/index.jsp, accessed on August 11, 2022), classifying the genes into biological process (BP), cellular component (CC), and molecular function (MF) categories. For the enrichment and localization analysis of DEGs throughout the tomato genome, the online platform ShinyGO 0.80 was used (http://bioinformatics.sdstate.edu/go/, accessed on September 19, 2022).

## Assignment of metabolic pathways

The assignment of metabolic pathways was performed using the Kyoto Encyclopedia of Genes and Genomes (KEGG) through the KEGG BlastKOALA annotation and mapping web server (https://www.kegg.jp/blastkoala/, accessed on August 9, 2022), using the taxonomic ID number 4081 corresponding to *S. lycopersicum*.

## SNP analysis

Reads obtained after mRNA-Seq were paired and mapped to the tomato reference genome (SL3.1) using BWA (Li & Durbin 2009). Duplicates were marked and removed using picardTools (https://broadinstitute.github.io/picard/). Variant calling was performed using samtools and bcftools (v0.1.19) (Li 2011). VCF files with variant detection were obtained through the bcftools mpileup command, and results were filtered with a quality value greater than 30. After alignment and variant calling, statistics, annotation, and graphs were generated in R and SRPlot (Tang et al. 2023). For the enrichment analysis and other complementary analysis, the online platform ShinyGO 0.80 was used (http://bioinformatics.sdstate.edu/go/, accessed on December 16, 2023).

## Differential gene expression with RT-qPCR

Down- and upregulated genes were selected for validating the correlation of the RNA-Seq data using RT-qPCR. Specific oligonucleotides were designed for three upregulated genes and two downregulated genes, involved in metabolic processes such as salicylic acid synthesis and plant development (Table S1). Each gene was evaluated in triplicate. The KAPPA SYBR® FAST One-Step qRT-PCR Master Mix (2x) kit was used following the manufacturer’s specifications, with a final volume of 10 µL per reaction. The amplification program was performed on the StepOnePlus™ Real-Time PCR System (Applied Biosystems, Thermo Scientific) using the following amplification program: 5 min at 42 °C, 5 min at 95 °C, 40 cycles of 5 min at 95 °C, and 30 s at 60 °C. Expression levels were calculated using the 2^(−ΔΔCt) method (Livak & Schmittgen 2001). Glyceraldehyde 3-phosphate dehydrogenase (GAPDH) from *S. lycopersicum* was used as an endogenous reference gene. Statistical analysis was performed using GraphPad Prism 8 GraphPad Software, California, USA (https://www.graphpad.com).

## Scanning *electron* microscopy for sample processing and imaging

Fresh tomato leaf samples were harvested from the analyzed plants. Subsequently, these samples were cut into approximately 0.5 cm^2^ squares and fixed in 2.5% glutaraldehyde within a 0.1 M phosphate buffer (PBS) at a pH of 7.4. Following fixation, three phosphate buffer washes were performed and a post-fixation step involving 1% osmium tetroxide dissolved in PBS. Dehydration of the tissue was meticulously conducted using a gradient of ethanol concentrations ranging from 50 to 100%. Samples underwent a critical point drying, were sputter-coated with gold, and were visualized utilizing the FE HRSEM Auriga 3916 scanning electron microscope. Finally, trichome number on the adaxial (upper) and abaxial (lower) surfaces of the leaves was determined. Statistical analyses were calculated using Student’s *t*-tests for parametric samples.

## Statistical analysis

The statistical analysis of cotyledon width and length was calculated using one-way ANOVA for non-parametric samples and analysis of plant length was calculated using Student’s *t*-tests for parametric samples. Photosynthetic rate measurement analyses were calculated using one-way ANOVA for parametric samples. Significant differences in relative expression values between the endogenous gene and the samples in RT-qPCR assays were calculated using Student’s *t*-tests for parametric samples.

## Results

### Colchicine-treated tomato seeds displayed vigorous phenotype

**Fig. 1:**
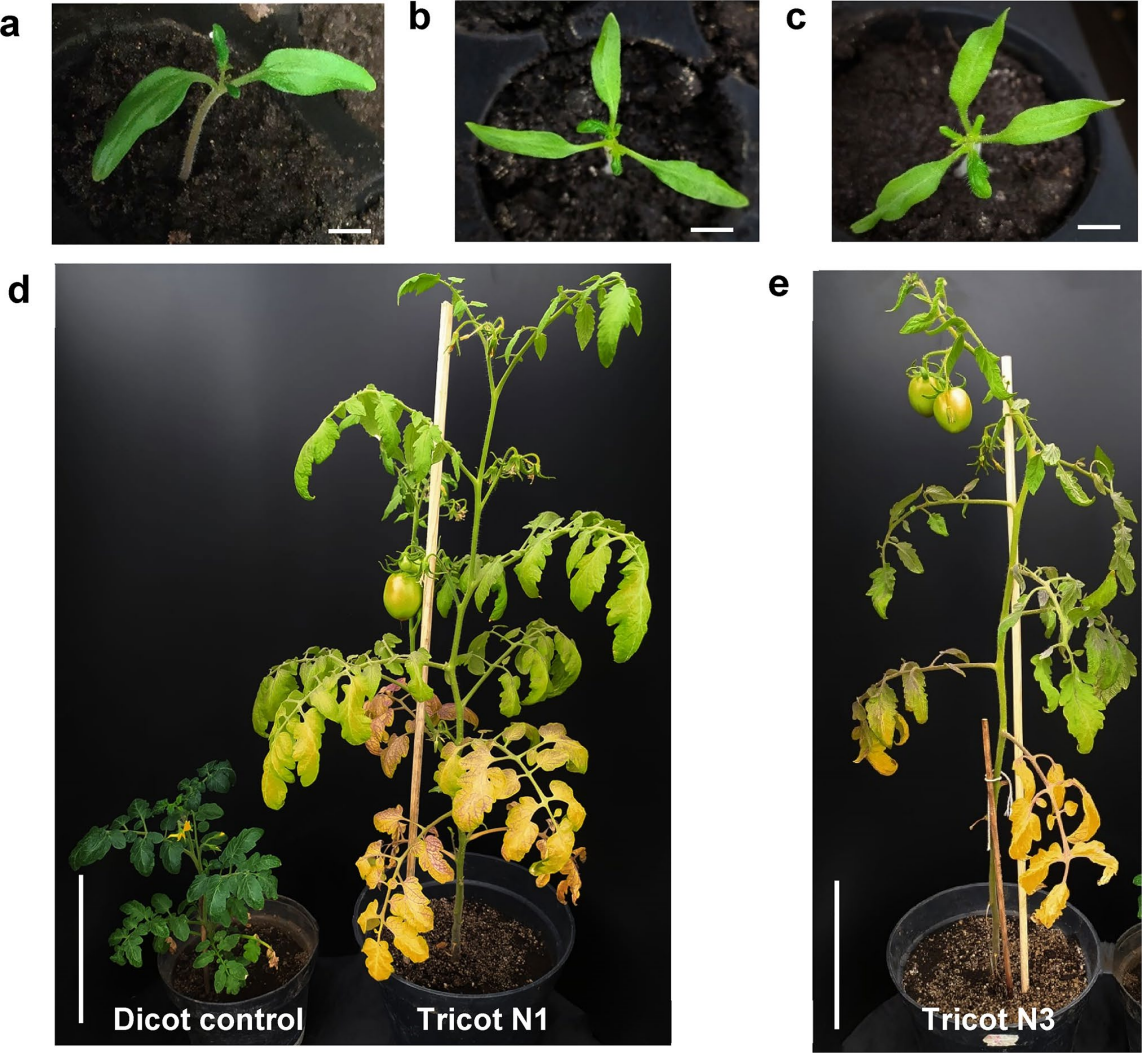
Morphology of Tricot tomato plants. Plantlets of (a) Dicot control, (b) Tricot N1, (c) Tricot N3. Three-month-old tomato of (d) Control, left and tricot N1. (e) Tricot N3. Note dicot control is starting flowering, while tricot N1 is already maturing tomato fruits. Bar in A, B and C is 1 cm. Bar in D and E is 15 cm

Tomato seeds treated with the antimitotic agent colchicine were germinated and plantlets were assessed based on their phenotype. The plants obtained after one month of growth were subjected to flow cytometry analysis to confirm the ploidy status from T0 to T2. Parental plants contained nuclei with 1.57 pg DNA content, while mixoploid J46 contained 1.50 pg, 3.01 pg DNA and tetraploid J53 duplicated the nuclei content. (Figure S1a). Filial segregation of tetraploid J53 tomato to T1 yield mixoploid plants, which maintained their DNA content in the next generations. The mixoploid J46 in contrast, maintained stable in terms of its DNA content for the three evaluated generations. (Figure S1b). No differences were registered in the stems and leaves shape compared to control parental plants. However, J46 changed its growth habit from apical dominance to bush growth (Figure S1d). Other analyses were conducted on T1 plants, potentially influenced by colchicine treatment, particularly focusing on chlorophyll content and photosynthetic rate. The mixoploid leaves exhibited a significant difference in chlorophyll content compared with the plant leaves of the control (Figure S1e). The results of the photosynthetic rate analysis demonstrated that colchicine-treated plants exhibited a statistically significant increase when compared to parental plants (Figure S1f). To assess the ploidy status of the T2 generation, extracted nuclei of these tomato lines were measured by flow cytometry, showing loss of mixoploid status, and resulting in diploid plants (Figure S1c). However, among the 165 obtained tomato seedlings of the T2 generation, five (3%) displayed three cotyledons with a similar length to the foliar blades present in control and dicot tomatoes, although with a greener color and pronounced curvature, shorter and thicker petioles, lanceolate foliar blades with smooth edges, and pinnate venation. Their phenology was observed over the course of three months, showing taller growth compared to the control tomato plants, and apical dominance was observed, in contrast to the bushy habit of control plants (Fig. 1a-d). Three independent tomato plants displaying tricotyledon morphology were assessed for nuclear DNA content, obtaining 1.62, 1.51 and 1.72 pg per nucleus, respectively, while control plants contained 1.60 pg per nucleus, although statistical comparison with a p-value of 0.05 did not find significant differences.

Other interesting morphological characteristics were affected by the colchicine treatment. The T2 of J53 plant exhibited an increase in trichomes on the abaxial leaf surface (Figure S2a). Furthermore, T3 plants of this tomato line maintained this phenotype, showing a heightened abundance of trichomes. In contrast, the adaxial side didn´t exhibit a significant difference (Figure S2b-e). The number of three cotyledons remained unchanged in T3 plants; however, there was a notable alteration in their size compared to the control, particularly in their width. This difference was statistically significant in the T3 plants of the tomato line that exhibited the tricotyledonous phenotype. Nevertheless, no significant difference was observed in the cotyledon length (Figure S3a, b). In contrast, the tricotyledon plants maintained an apical dominance phenotype compared to the control plants (Fig. 1d). Additionally, the progeny of tricot N1 plant also maintained this phenotype; in contrast, the progeny of tricot N3 plant didn´t display apical dominance in comparison with the control plants (Figure S3d, e). Finally, the progeny of tricot N3 (tricot N3.1) plant showed an increased photosynthetic rate compared to the control with statistical significance (P < 0.0001) (Figure S3c). The vigorous growth of three-month-old tricot tomato is observed when compared to control plants (Fig. 1). To note is the dicot control plant is starting flowering, while tricot N1 has flowered and is already maturing tomato fruits. The early plant development, including flowering and fruit development, was remarkable in tricot N3 plant.

## Transcriptome assembly and data acquisition

Two T2 independent plants with the tricotyledonous phenotype were selected for further transcriptome analysis (tricot N1 and N3) (Fig. 1b, c). Raw data for each tricot were independently analyzed, considering they were produced from different colchicine-treated seeds. The RNA-Seq data have been deposited in the Short Read Archive (SRA) of the National Center for Biotechnology Information (NCBI) with accession number PRJNA1108447. High-quality pair-end raw sequences comprised 25 million reads of control plant and 21 and 33 million reads for each tricotyledon plant.

## Identification and analysis of differentially expressed genes (DEGs)

**Fig. 2:**
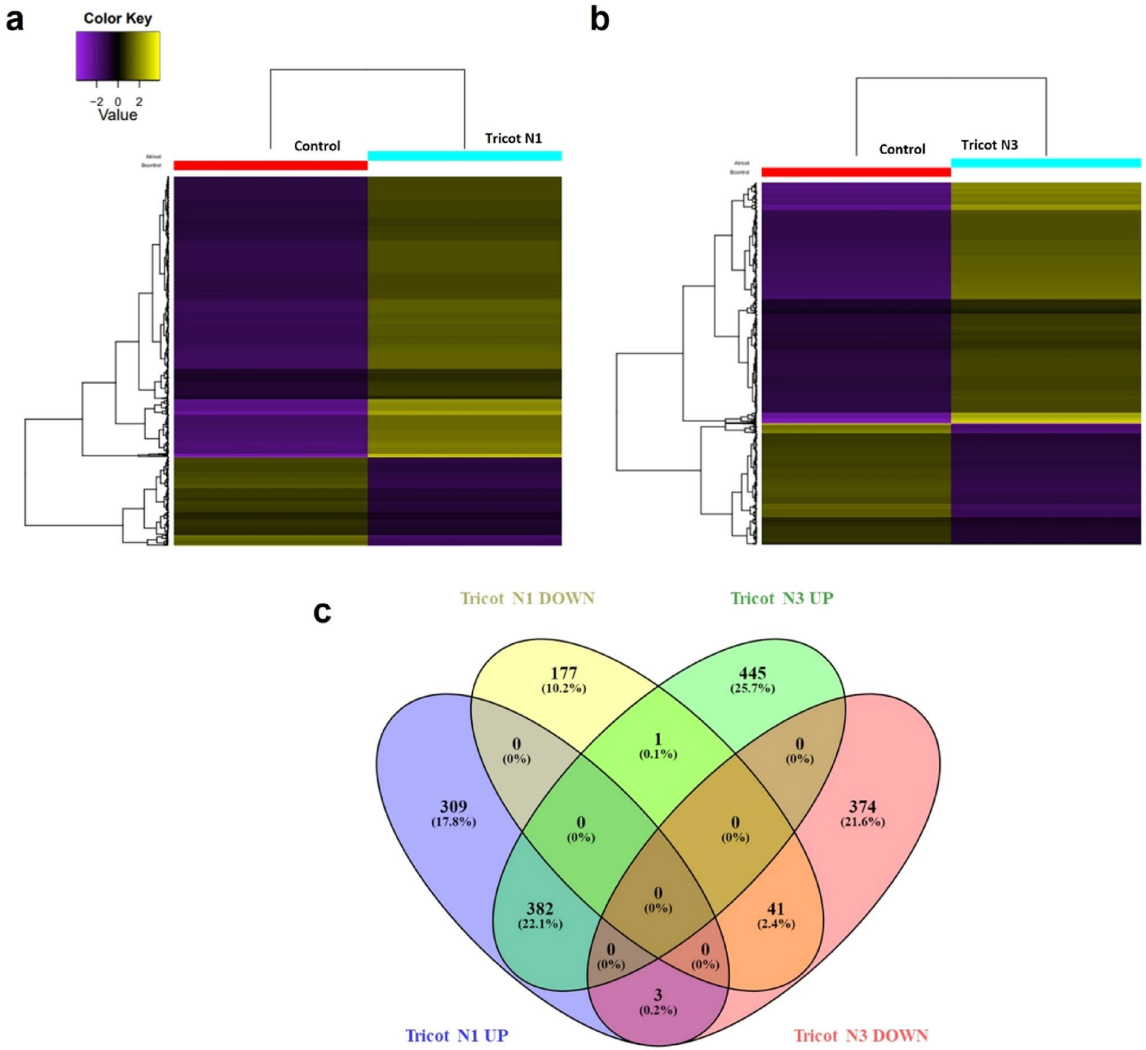
Differential gene expression in tricot tomato plants. (a) Heatmap of differentially expressed genes in tricot N1 tomato plant vs control plant, (b) Heat map of differentially expressed genes in tricot N3 tomato plant vs control plant. Upregulated transcripts are shown in yellow and the repressed ones in purple in tricot tomato plants (right columns) vs. control (left). Dendrogram represents genes with similar expression levels. (c) Venn diagram showing overlap in upregulated and downregulated genes in N1 and N3 plants. Downregulated genes from N1 plants are shown in yellow, upregulated genes of tricot N1 plants are shown in purple, N3 upregulated genes are shown in green, and the N3 downregulated genes are shown in red

Transcriptomic analysis from tricot N1 and N3 plants revealed 913 and 1246 differentially expressed genes (DEGs), respectively, relative to untreated tomato control plants. The heatmap obtained from this analysis (Fig. 2a, b) shows gene expression profiles, allowing the clustering of transcripts with similar expression levels in tricotyledonous plants. The Venn diagram of the DEGs in the tricotyledonous plants showed that there are 382 overexpressed genes, and 41 suppressed genes shared by N1 and N3 plants. It is important to consider the limitations of the transcriptome analyses, although its potential to identify differential transcript accumulation, those mRNAs still need to be translated to proteins. As for the case of metabolite accumulation, enzymes encoded in overexpressed transcripts need to be part of secondary metabolism for the synthesis of such low molecular weight molecules. Taking this consideration into account, the transcriptome analyses suggest the possible molecular mechanisms associated to the observed phenotype in the colchicine treated plants (Fig. 2c), yet to be confirmed by proteome and metabolome analyses.

Mapping of the N1 and N3 common overexpressed genes revealed that they were distributed across all *S. lycopersicum* chromosomes. However, four regions in chromosome 2 had a statistically higher number of such genes (Figure S4). A fold-change greater than 4 indicates a significant difference in gene expression (Spang, n.d.) and as the fold-change level reaches ≥ 2 the number of differentially expressed genes decreases significantly; however, it could be expected that these have a greater significance in this particular biological process, i.e. the observed growth enhancement as a result of colchicine treatment (Dalman et al. 2012). Overexpressed genes with a fold-change value > 4 was distributed in all chromosomes in tricotyledonous plants (Figure S5). Chromosomes 3 and 4 had the highest number of overexpressed genes, with a fold-change greater than 4 in both N1 and N3 plants. In contrast, chromosome 9 exhibited a lower number of genes showing differential expression. Genes with the greatest increase in expression of each tricot plant, such as SPX domain-containing protein 3, ascorbate oxidase, psi14C protein, auxin-regulated protein, LRR receptor-like serine/threonine-protein kinase At3g47570, arginine decarboxylase and NAC domain-containing protein 22, exhibited a significant increase in their fold-change value in both plants.

## Annotation and functional classification by Gene Ontology (GO)

**Fig. 3:**
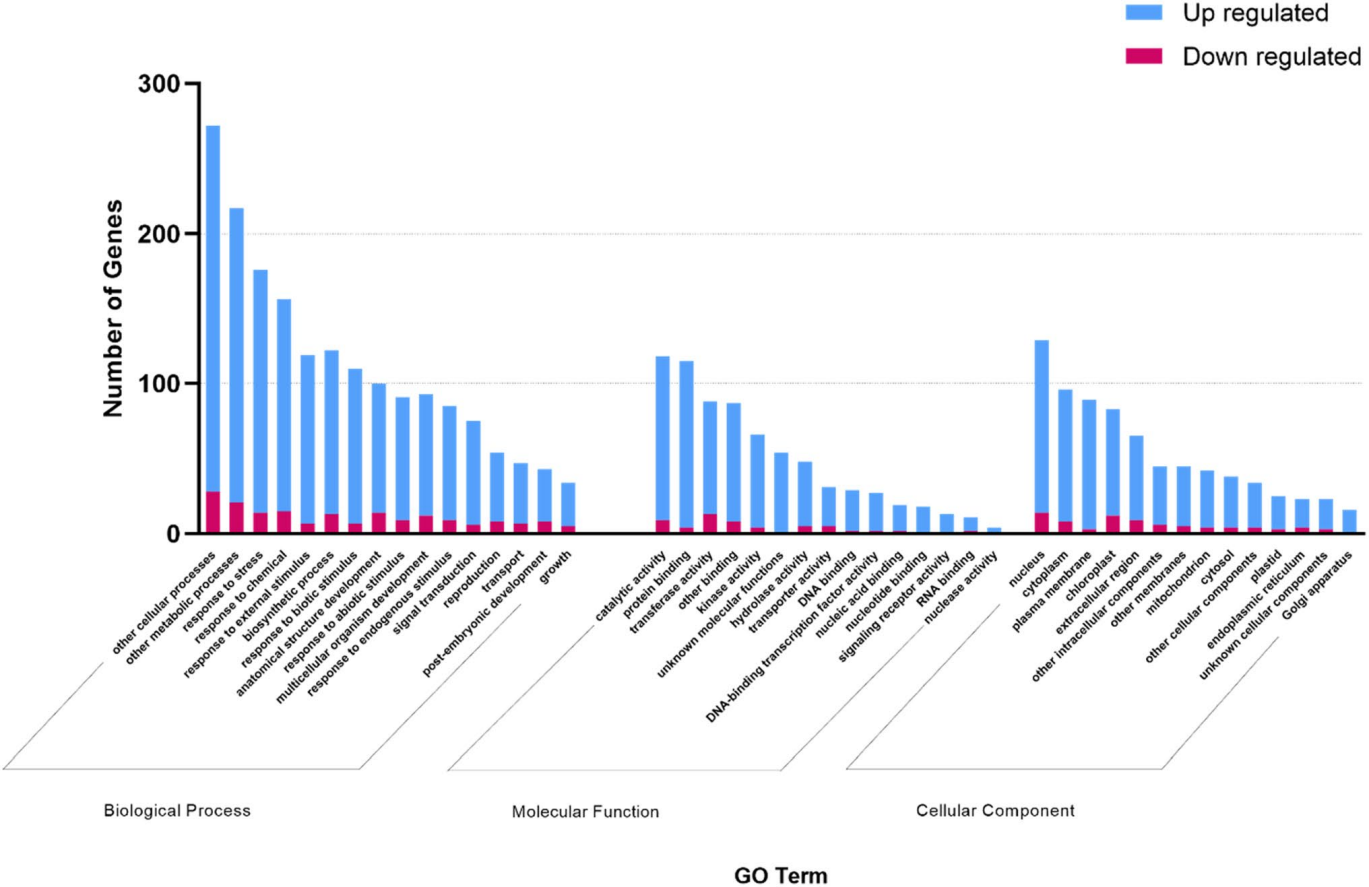
Gene ontology classification of DEGs between tomato tricot and dicot control plants. Differential genes were categorized by Biological Process, Molecular Function and Cellular Component. Blue represents upregulated and red color down regulated transcripts

To elucidate the biological functions of DEGs, a gene ontology classification analysis was conducted. The common DEGs for tricot N1 and tricot N3 plants were selected for further investigation. Using *A. thaliana* as reference, DEGs were classified into the categories of biological process (BP), cellular component (CC), and molecular function (MF) (Fig. 3). In each functional cluster, the top categories that are most prominently represented among the DEGs were shown. MF-related DEGs, such as those involved in catalytic and kinase activity, protein binding, transferase activity, and others were predominant. CC analysis showed enrichment in the nucleus, cytoplasm, plasma membrane, chloroplast, and extracellular region. Finally, BP analysis showed genes belonging to additional cellular processes, other metabolic processes, response to stress, response to chemical, response to external stimulus, biosynthetic process, and response to biotic stimulus. The GO analysis for each classification and their representative accessions showed a predominance of upregulated genes, compared to downregulated genes (Fig. 3).

## Gene enrichment analysis by PlantGSEA and KEGG metabolic pathway assignment

**Fig. 4:**
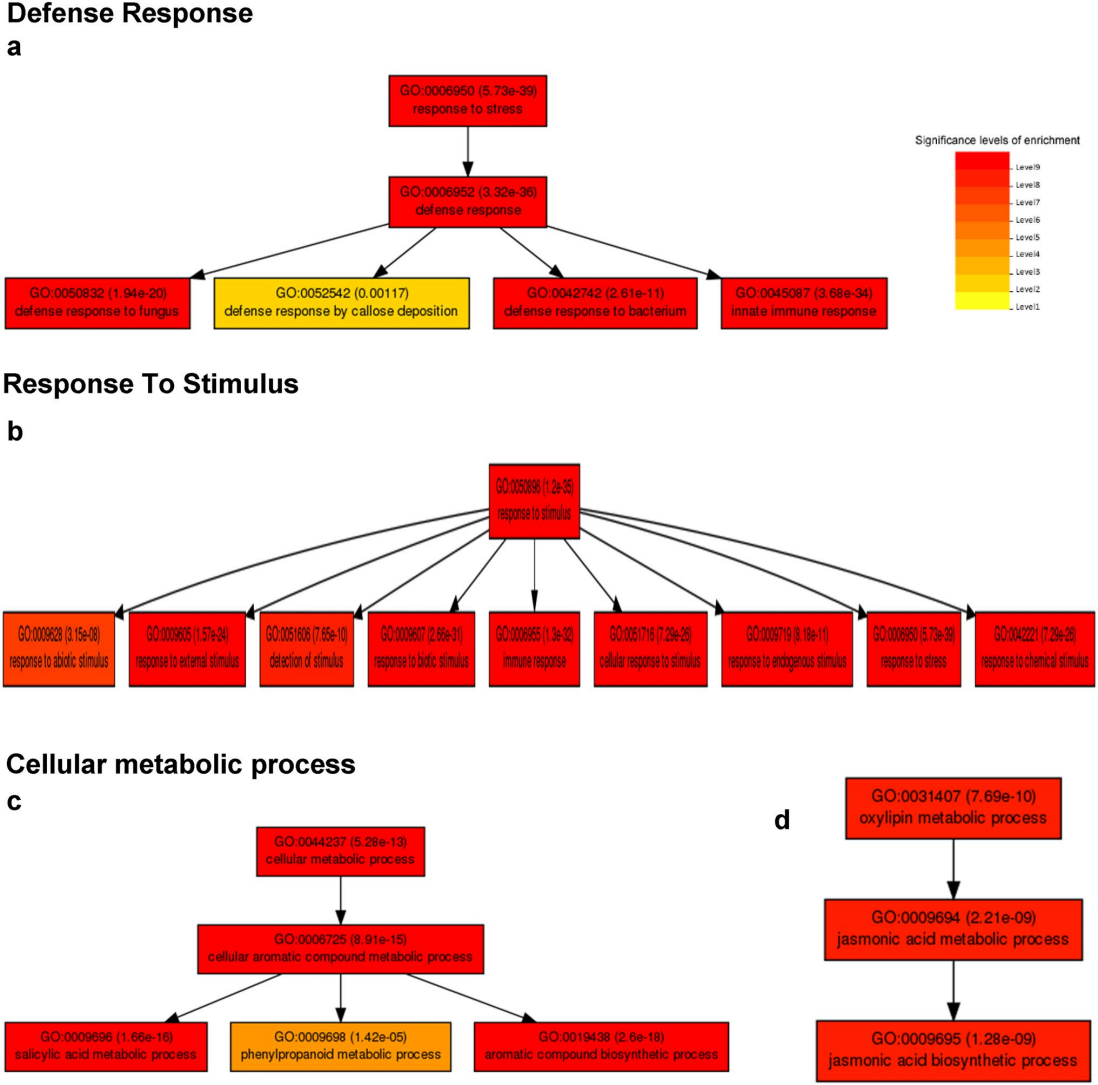
Hierarchy analysis of differentially overexpressed transcripts in GO terms of biological processes by Plant GSEA. (a) Defense response, (b) Response to stimulus, (c) Metabolic process, (d) Metabolic process involving jasmonate. Significant levels of enrichment are shown in a gradient from red (higher levels) to yellow (lower levels)

The metabolic pathways in which the products encoded by the DEGs participate were identified. The enrichment of the overexpressed genes using PlantGSEA revealed changes in biological processes with a higher number of genes, including the following: (1) defense response, (2) response to stimulus, (3) cellular metabolic processes and (4) response to hormone stimulus (Fig. 4). The highest statistically significant enrichment levels were observed in the gene set for response to stress (GO:0006950), response to stimulus (GO:0050896), cellular metabolic process (GO:0044237), response to organic substances (GO:0010033) and response to endogenous stimulus (GO:0009719). Within the stress response gene set (GO:0006950), defense response to fungus, defense response to bacterium, and innate immune response were enriched (Fig. 4a). In the response to stimulus gene set (GO:0050896), transcripts involved in biotic and abiotic stimulus response, stimulus detection, stress response, and chemical stimulus response (Fig. 4b). The metabolism of aromatic compounds, synthesis, and metabolism of salicylic acid, and jasmonate metabolism were significantly altered pathways where primary metabolism pathways (GO:0044237), response to hormone stimulus (GO:0009725) and response to organic substances (GO:0010033) converge (Fig. 4d, e).

Among the upregulated transcripts, 217 genes were assigned in the cotyledon ontology (PO:0020030), showing a higher enrichment in biological processes such as stilbenoid, diarylheptanoid and gingerol biosynthesis, flavonoid biosynthesis, ether lipid metabolism, and glycerophospholipid metabolism (Figure S6).

The results of the KEGG analysis of the overexpressed genes showed differences in various metabolic pathways. Three pathways were selected based on their importance level, considering beneficial traits such as resistance to pests and diseases, enhanced organoleptic characteristics in plants, and the number of involved genes: 1) plant-pathogen interaction, 2) plant hormone signal transduction, and 3) flavonoid biosynthesis. Calcium-dependent protein kinase (CDPK), cyclic nucleotide gated channels (CNGCs), calmodulin and calmodulin-like (CaM/CML), pathogenesis related genes transcriptional activator (Pti5), mitogen activated protein kinase 3, WRKY transcription factor 25 (WRKY25), ethylene-inducing xylanase ½ (EIX1/2) and pathogenesis related protein 1 (PR1) were upregulated in the plant-pathogen interaction pathway of the T2 tricotyledonous tomato plants (Figure S7). On the other hand, ethylene-responsive transcription factor 1 (ERF1), jasmonate ZIM domain-containing protein (JAZ), and protein phosphatase 2C (PP2C) were upregulated in the plant hormone signal transduction pathway (Figure S8). Finally, the shikimate O-hydroxy-cinnamoyl transferase, caffeoyl-CoA O-methyltransferase and naringenin 3-dioxygenase enzymes were overexpressed in the flavonoid biosynthesis pathway (Figure S9). The downregulated genes were grouped into 22 enriched pathways, with notable pathways including secondary metabolite biosynthesis and carbon metabolism.

## SNP analysis

**Fig. 5:**
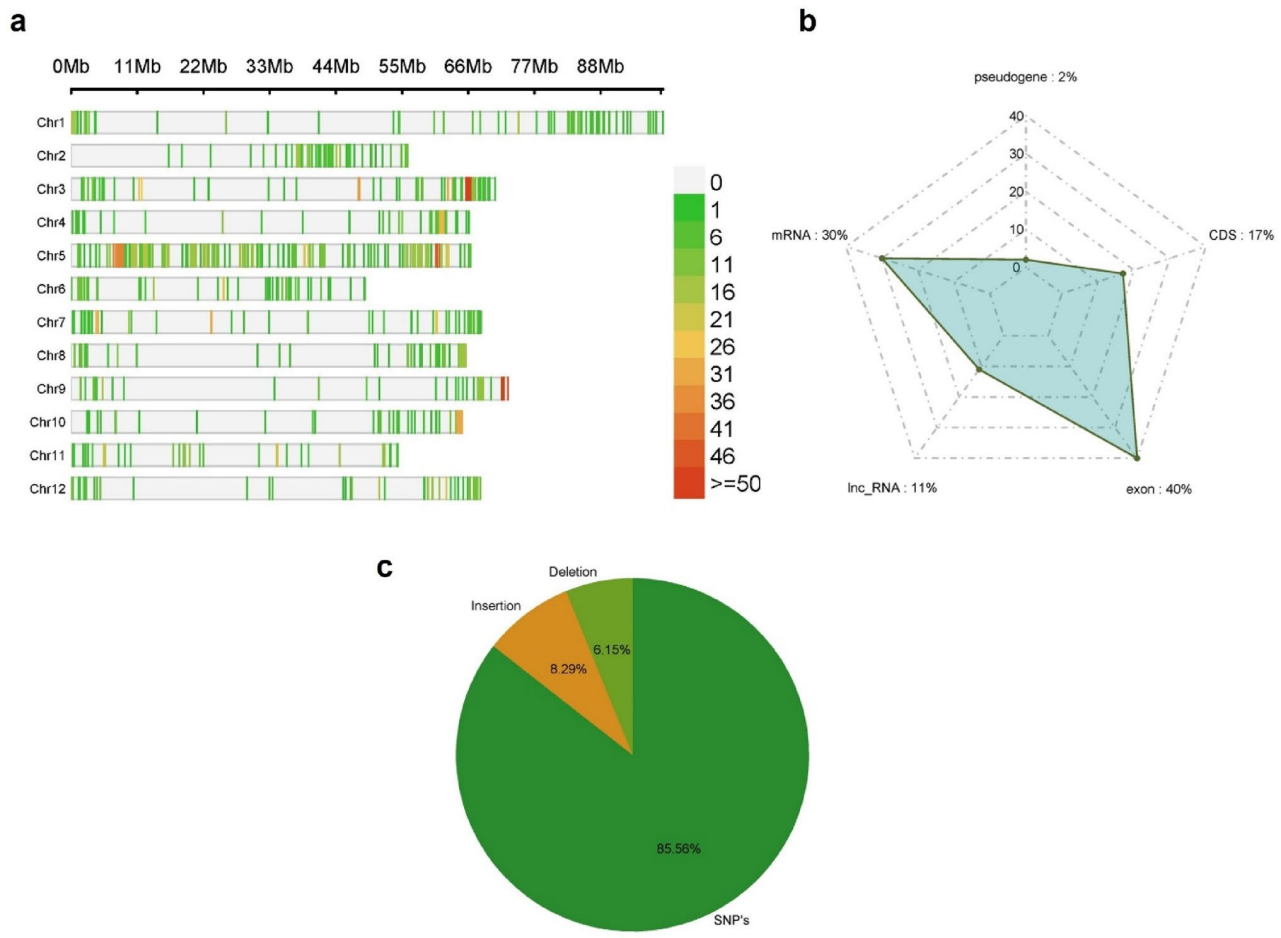
Single SNPs between tricot N1 and tricot N3 compared to control. (a) Single Nucleotide Polymorphism (SNP) density plot across the chromosomes of tomato representing the number of SNPs within 1 Mb window size; the different colors represent the density. (b) Classification of SNPs. (c) Percentage of indels (insertion, deletion, and SNP’s)

SNP/indel analysis was conducted using transcript sequences, with unique variants identified in the tricot plants compared to the control reference. A total of 2032 SNPs, 197 insertions, and 146 deletions were identified, constituting 85.56%, 8.29%, and 6.15% of the variations, respectively (Fig. 5c). These filtered SNPs provide coverage throughout the 12 chromosomes of tomato, all of these were widely distributed across the genome, covering the initial and final region of the chromosomes, and a minor density in the centromere region (Fig. 5a). The chromosome in which the highest density of these was observed was chromosome 5, while the one with the lowest density was chromosome 8. The identified SNPs were classified into exon, coding regions (40%), while the remaining percentage is classified into mRNA, long noncoding RNA (lncRNA), CDS and pseudogene, representing 30%, 11%, 17% and 2% respectively (Fig. 5b). The genes to which these variants belong were found to be enriched in metabolic pathways such as glycosphingolipid biosynthesis-globo and isoglobo series, lysine degradation, N-glycan biosynthesis, pyruvate metabolism, glycolysis/gluconeogenesis, TCA cycle, and others (Fig. S10).

## Analysis of DEG by quantitative RT-PCR

To assess differential expression using an alternative technique, genes with the highest fold-change values associated with salicylic acid metabolism (upregulated) and plant development (downregulated) were selected for quantitative RT-PCR analysis (Table 1).

**Table 1:** Selected transcripts for differential gene expression by RT-qPCR

Upregulated genes		FC Tricot N1	FC Tricot N3
1	XM_026031856.1 PREDICTED: *Solanum lycopersicum* probably inactive leucine-rich repeat receptor-like protein kinase At5g48380	4.4725	5.4083
2	XM_004238128.4_F PREDICTED: *Solanum lycopersicum* patatin-like protein 3	2.1546	3.7576
3	NM_001247904.2_F *Solanum lycopersicum* allene oxide synthase (aos), mRNA	6.1983	3.3340
**Downregulated genes**		**FC** **Tricot N1**	**FC** **Tricot N3**
1.1	NM_001251869.2_ R *Solanum lycopersicum* late embryogenesis abundant protein	−4.5653	−5.3214
2.1	XM_010323527.3_R PREDICTED: *Solanum lycopersicum* putative lysine-specific demethylase JMJ16	−2.0234	−2.4038

FC: Fold-Change

**Fig. 6:**
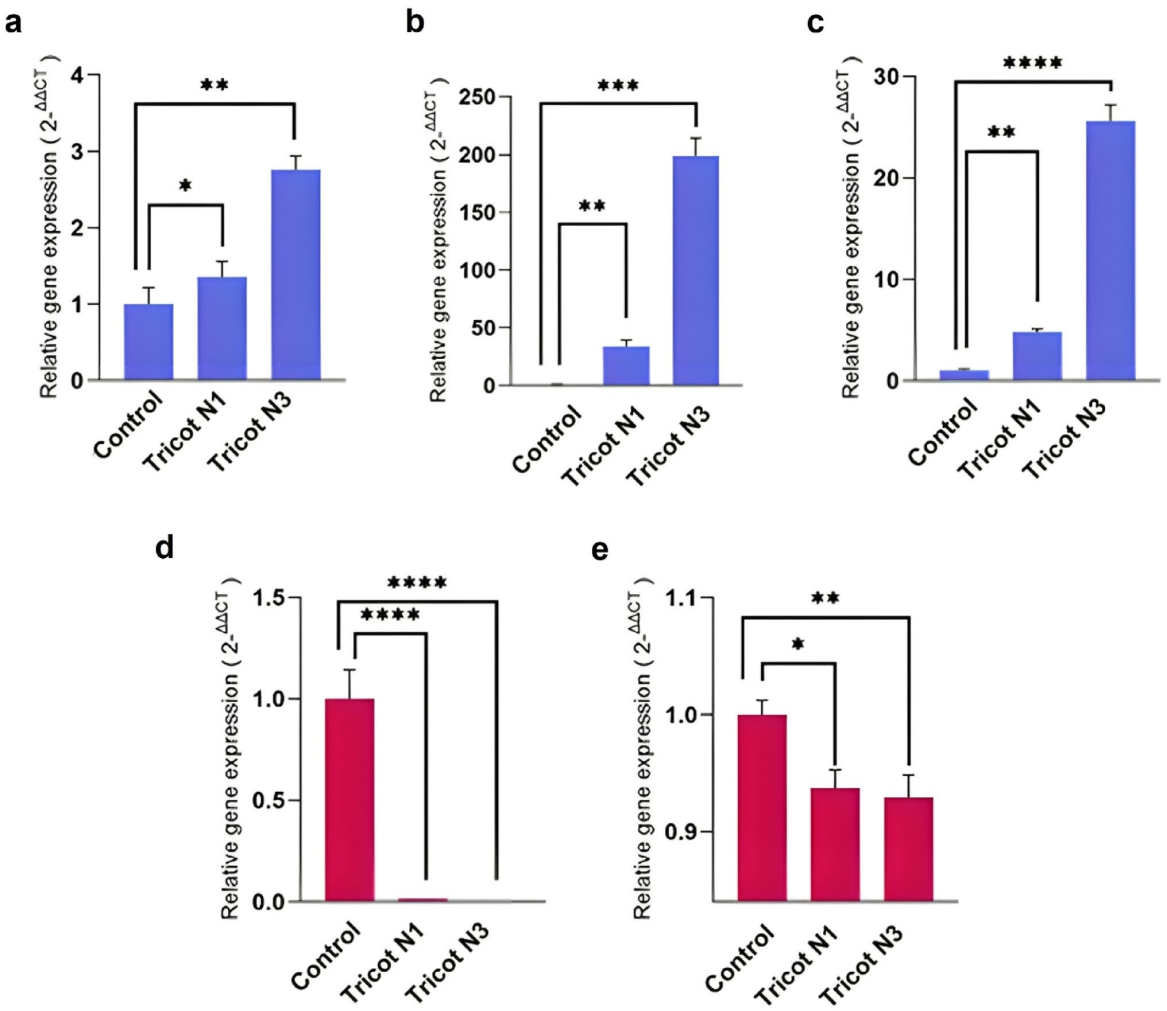
Differential gene expression by RT-qPCR of tricot tomato plants and control. (a) inactive leucine-rich repeat receptor-like protein kinase (*Arabidopsis* ID At5g48380), (b) patatin-like protein, (c) allene oxide synthase (aos), (d) late embryogenesis abundant protein, (e) putative lysine-specific demethylase JMJ16. The graphs in blue correspond to the overexpressed genes and those in red to the suppressed genes. The bars represent the standard deviation. Asterisks reflect statistical significance * P ≤ 0.05, *** P ≤ 0.001, **** P ≤ 0.0001

The results showed that the three upregulated transcripts patatin-like protein, inactive leucine-rich repeat receptor-like protein kinase At5g48380, and allene oxide synthase (AOS), were upregulated in tricotyledonous plants, consistent with the RNA-seq data. The selected downregulated genes, late embryogenesis abundant protein (LEA) and putative lysine-specific demethylase (JMJ16), showed that both were suppressed in tricotyledonous plants (Fig. 6). On the other hand, there was a difference in gene expression levels in tricotyledonous plants. N3 plant showed significant difference in most of the genes evaluated such as the kinase Atg48380, patatin like protein, LEA and JMJ16 demethylase, compared to the control and N1. The results obtained for the set of genes confirmed the data obtained from the high-throughput RNA sequencing analysis of the RNA populations.

## Discussion

The antimitotic agent colchicine has been widely and successfully used to increase genetic diversity and thus crop improvement to enhance tolerance to pathogens, pests, or stress in various species through polyploidization, (Zhu et al. 2021). Polyploidy leads to various phenotypic changes in plants, such as enlargement of organs and increased cell size, alteration in biomass, and cell wall composition (Corneillie et al. 2019). Furthermore, an increase in trichome number and size is observed in colchicine-treated tetraploid plants (Mohammadi et al. 2023). Polyploidization can also induce changes in chlorophyll content and photosynthetic rate, which correlates with the amount of DNA per cell. Plants with a higher level of ploidy tend to have a larger cell size or more cells per leaf unit; indeed, if there is an increase in leaf area which is not proportional to the increase in cell volume, it could lead to greater photosynthetic efficiency. Mixoploid plants have cells with different chromosome numbers which can increase the size of the cells, and these often have a greater photosynthetic capacity (Warner & Edwards 1993).

In the present work tomato plants were treated with colchicine with the aim of obtaining polyploid plants with increased vigor resulting from enhanced genetic diversity. However, plants precisely showing this trait, in terms of improved growth relative to controls, were not polyploid. Instead, these were chimeras, or mixoploids, in the sense that only some cells or tissues were effectively tetraploid. Additionally, these plants harbored several point mutations throughout the genome. While the mechanism through which colchicine induces polyploidization has been studied thoroughly, i.e. by blocking segregation of chromosomes during meiosis via microtubule depolymerization (Caperta et al. 2006; Shu et al. 2012), how does this chemical agent induce single nucleotide mutations is largely unknown. Indeed, colchicine and other chemical mutagens can induce single nucleotide polymorphisms (SNPs), and these potential changes can serve as a source of genetic diversity (Shu et al. 2012). The impact of colchicine is not confined to genetic hotspots, rather, it appears to induce mutations throughout the whole genome (Kuo et al. 2020; Damon 1957). It has been observed that the level of gene expression tends to increase with the number of copies of genes or chromosomes in cases of aneuploidies. Moreover, a simple increase in the number of copies of entire chromosomes or portions can lead to mutant phenotypes (Kojima & Cimini 2019; Prelich 2012). Colchicine treatment can also lead to changes in gene expression in polyploid plants, with a high enrichment of gene ontology (GO) terms such as catalytic activity, membrane activity and transporters, phenylpropanoid biosynthesis pathway, phenylalanine metabolism, plant hormone signaling transduction, and starch and sucrose metabolism (Zhou et al. 2017).

While N1 and N3 plants were not truly polyploid, their phenotype suggested extensive genetic modifications. Indeed, SNP analysis indicated the presence of more than 2000-point mutations throughout the genome of these plants. Interestingly, the majority of these fell relatively close to both ends of the chromosomes, although SNPs were roughly evenly distributed along the length of chromosome 5. Also, annotation of SNPs indicated that one of the most enriched metabolic pathways was the biosynthesis of glycosphingolipids, which are essential for the integrity of the plasma membrane, tolerance, and response to various biotic and abiotic stresses, as well as cell signaling (Haslam & Feussner 2022).

An additional trait observed after this treatment was the emergence of three cotyledons in treated plants. Modifications in cotyledon leaf number or development is a phenotype that rarely occurs in dicotyledonous plants, and there is limited information regarding the occurrence of this trait in tomato. In 1943, Reynard examined 497 tomato varieties and found that approximately 1.7% of the analyzed seedlings exhibited a polycotyledonous phenotype, although it was also reported that these plants did not show a significant increase in the number of leaves or fruits.

Cotyledon development is a crucial process in plant embryogenesis that determines the structure and function of the seedlings. The cotyledon is the first organ to differentiate during embryogenesis, which is responsible for supporting the growth of the plant until the seedling is fully developed and photosynthetically competent (Madishetty et al. 2006). Cotyledons are transient structures that can persist for a certain time after emergence, or they can be retained by the plant throughout its vegetative life (Chandler 2008); having a larger number of cotyledons in a species is referred to as polycotyly. This process is tightly regulated and involves multiple molecular pathways, including transcriptional regulation, hormone signaling, and cell differentiation (Al-Hammadi et al. 2003). The size, shape, and number of cotyledons can vary between plant species and even within the same plant depending on environmental cues. However, as described previously, these plants showed not only an overall normal morphology but were more vigorous and showed a larger size than untreated controls. Thus, the presence of an extra cotyledon did not hamper its normal development. More work will be required to determine whether this novel morphological feature is associated with growth enhancement.

It is reasonable to assume that the combination of the mutations induced by colchicine, even when not resulting in polyploidy, causes the observed increase in growth of the N1 and N3 plants, which in turn causes differential expression of a set of genes. Auxin is an important regulator of cotyledon development (Chandler 2008). Arabidopsis with defective auxin transport display abnormalities in cotyledon formation, and the pinoid1 (pd1) mutant show alterations in the number, shape, and size of cotyledons (Zhao 2010). Similarly, in a polycotyledonous mutant of tomato cv. Ailsa Craig, increased polar auxin transport was observed and the phenotype of this plant may have been influenced by altered auxin distribution (Al-Hammadi et al. 2003). Thus, it could be speculated that auxin function may be dysregulated in N1 and N3 plants; however, only one differentially expressed gene in both is linked directly to auxin function (NM_001247706.1; encodes an auxin-regulated protein). On the other hand, flavonoids modulate auxin transport by regulating the activity of auxin-transporting P-glycoproteins, as well as the activity of regulators such as phosphatases and kinases (Peer & Murphy 2007). Flavonoids can also regulate auxin transport by inhibiting auxin efflux, while also modulating the activity of auxin-responsive genes (Mierziak et al. 2014). In addition, flavonoids can act as cofactors for auxin signaling, enhancing the stability and activity of the Aux/IAA proteins that regulate gene expression (Brown et al. 2001; Ringli et al. 2008). Consistent with this, several genes related to flavonoid biosynthesis are upregulated in N1 and N3 plants (Fig. S9), suggesting a link with altered cotyledon number of these plants. Phenylpropanoid biosynthesis may be also enhanced in N1 and N3 plants, given that genes for shikimate O-hydroxycinnamoyl transferase and coenzyme caffeoyl-CoA O-methyltransferase are upregulated. These play a crucial role both lignin biosynthesis and composition during plant development; indeed, alterations in lignin levels directly impact plant development (Dong et al. 2022). Lignin plays a crucial role in reinforcing plant structure and defense against pathogens and mechanical damage. Therefore, overexpressing genes regulating flavonoid biosynthesis in tomato plants could potentially enhance anthocyanin and lignin content.

Some upregulated genes that exhibited a high FC in both tricotyledonous plants have a role in phosphate signaling, which is essential for plant growth and development, such as the SPX domain protein, and an inorganic pyrophosphatase, which is also involved in several abiotic stress-related processes (Secco et al. 2012; Wang et al. 2019).

Differentially expressed genes in N1 and N3 plants were found in all chromosomes. However, four regions of chromosome 2 were enriched in such genes compared to other parts of this chromosome. Genes located in these regions form part of the plant-pathogen interaction and stress response pathways. Among these are the *WRKY53* gene, which its overexpression can delay leaf senescence; and *WRKY70*, which is involved in brassinosteroid-regulated plant defense, growth and negative regulation of drought tolerance (Zentgraf & Doll 2019; Li et al. 2004, 2020). Overexpression of yet another one, *WRKY40*, in transgenic tobacco and lemon enhances salt tolerance, while its silencing increases susceptibility to salt stress (Dai et al. 2018). Upregulation of these genes may be involved thus in certain phenotypic changes observed in tricotyledonous plants.

A differentially expressed gene found is *Pti5*, a transcription factor involved in tomato fruit ripening, which when overexpressed enhances resistance to pathogenic bacteria and accelerates fruit ripening (Wang et al. 2021). Another differentially expressed gene with a potentially related function common to N1 and N3 plants codes for a Leucine-rich receptor kinase (XM_010319127.3). This is homologous to the MALE DISCOVERER 1 protein in Arabidopsis, which plays a crucial role in pollen tube guidance (Wang et al. 2016). This protein is also involved in plant immunity to bacteria (Hou et al. 2021). One of the genes selected for validation of RNAseq analysis, homologous to the BAK interacting receptor kinase BIR from Arabidopsis (probably inactive leucine-rich repeat receptor-like protein kinase At5g48380; XM_026031856.1) is a negative regulator of innate immunity, but recent studies have shown to have a role in antiviral defense (Guzmán‐Benito et al. 2019).

Upregulation of the *ERF1, JAZ, MPK3* and *MPKKK5* genes (XM_004236637.4; NM_001247954.1; NM_001247431.2), whose homologs mediate the response to ethylene, jasmonate and the latter two to pathogens, respectively, may provide insights into the phenotype of colchicine treated plants. *ERF* is a key regulators in abiotic stress tolerance, such as salt, drought, and cold stress (Müller & Munné-Bosch 2015). Furthermore, upregulation of a gene for allene oxide synthase (NM_001247904.2), involved in synthesis of jasmonate, supports the notion that this response may be upregulated in N1 and N3 plants. Jasmonate is involved in plant defense, development, and various other physiological processes, including stamen growth, senescence, root growth, and the production of metabolites such as phytoalexins and terpenoids (Gomi 2020). Interestingly, the JMJ16 homolog, used for RNAseq validation and which was upregulated in N1 and N3 plants (XM_010323527.3), is a histone demethylase that, in *Arabidopsis*, suppresses leaf senescence. Furthermore, MPK3 and MPK6 are central to the pattern-recognition defense response, and, interestingly, MPKKK5 phosphorylates MPK3 in *edr1* mutants in Arabidopsis, helping to establish a defense response in this genetic background (Wang et al. 2023; Fernández-Milmanda 2023). It must be mentioned that the actual function of these genes in tomato has not been established; however, the tomato Clade VI lectin receptor kinase (NM_001320919.1) has been shown to function in resistance against *Phytophthora* (Wang et al. 2015), suggesting that the aforementioned genes may have a similar role in this plant. These findings suggest that the overexpression of these genes in tricotyledonous tomato plants may result in enhanced tolerance to biotic and abiotic stress.

Some upregulated genes in tricot N3 are involved in the trichome development, such as trichome birefringence-like 39 (XM_004243358) that belongs to the trichome birefringence-like protein family engaged in the O-acetylation of cell wall polysaccharides (Tian et al. 2021). The loss of some of these genes decreases the number of xylem vessels and slow development in plants as well (Yuan et al. 2016; Li et al. 2023). Also, the MYB13-like transcription factors upregulated in both tricotyledonous plants, and the AN2 MYB factor in upregulated in N3 control leaf trichome formation and development regulation (Chang et al. 2024), and overexpression of some *MYB* genes lead to an increase in trichome density (Wyrzykowska et al. 2021). Given that jasmonate regulates trichome development and density in tomato, specifically the glandular morphology (Chen et al. 2018), the nature of the upregulated genes in N1 and N3 plants could help explain the observed increase in trichome number and density in these plants.

In the present work we have shown that colchicine treatment in tomato resulted in plants that were mixoploid and showed a more vigorous growth than wild type ones. Mutations were roughly evenly distributed throughout all chromosomes, although biased toward chromosomal ends plants. Some of these mutations fell within regulatory and coding regions of genes and caused the emergence of an extra cotyledon in two independent sets of plants. Additionally, these mutations resulted in the upregulation and downregulation of sets of genes, which could be involved in the observed phenotype of mixoploid plants. Further analysis of the altered regulation of these genes will provide valuable information for future plant breeding studies.

## Supplementary Information

Below is the link to the electronic supplementary material.

Supplementary file1 (DOCX 4598 KB)

## Data Availability

The author responsible for distribution of materials integral to the findings presented in this article in accordance with the policy described in the Instructions for Authors (https://academic.oup.com/plcell/pages/General-Instructions) is: Beatriz Xoconostle-Cazares (bxoconos@cinvestav.mx).
