# Supplementary material for: Plant growth Enhancement in Colchicine-Treated Tomato Seeds without Polyploidy Induction
Source: Plant Mol Biol. 2024 Dec 12;115(1):3. doi: 10.1007/s11103-024-01521-1 (PMC11638462; doi:10.1007/s11103-024-01521-1)
Supplement: Supplementary file 1 — Supplementary file1 (DOCX 4598 KB) [file 11103_2024_1521_MOESM1_ESM.docx]

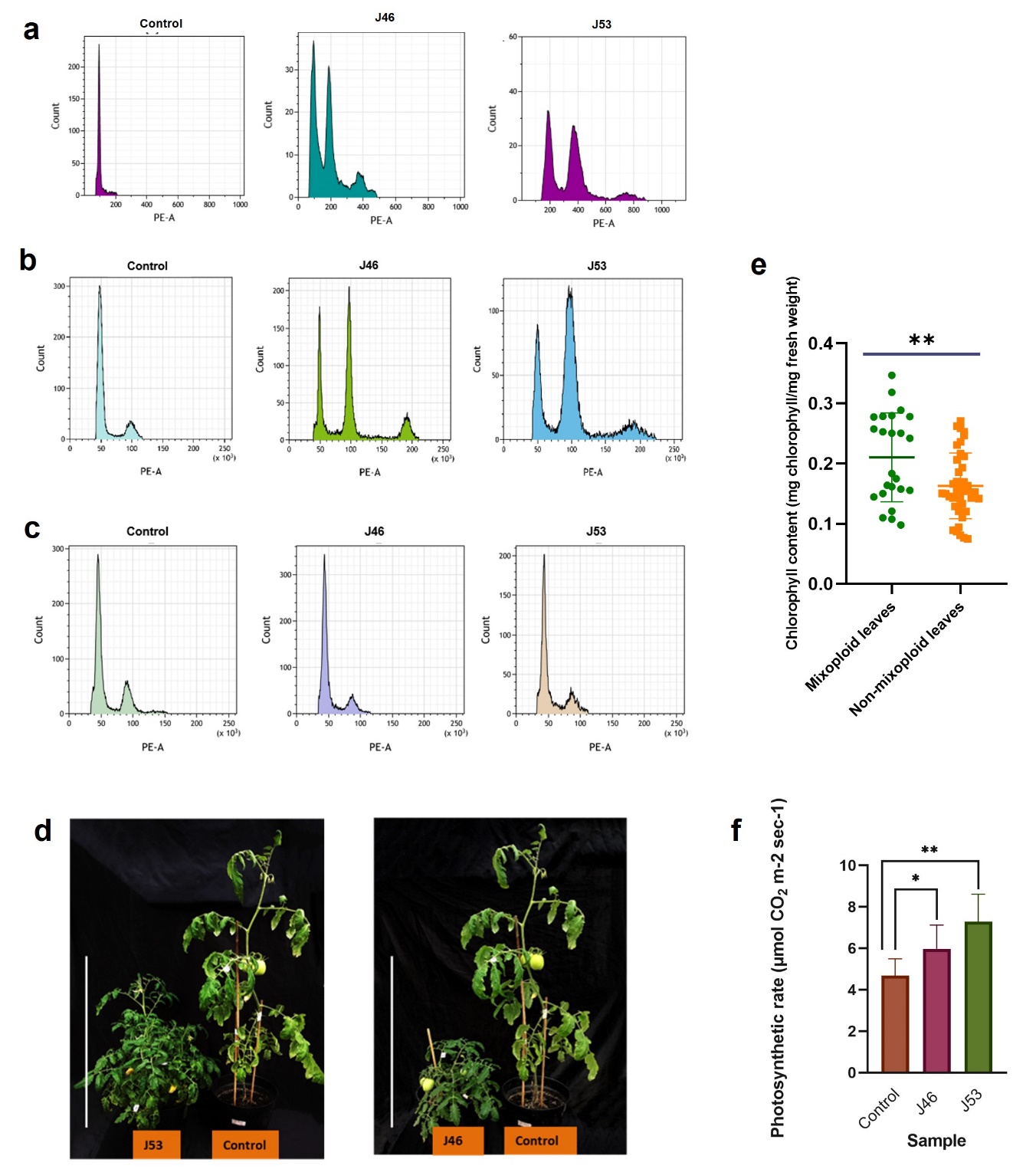


**Figure S1. Characterization of tomato plants treated with colchicine.** (a) Histograms of tomato T0 plants and control after treatment with colchicine 0.1% 48 h, (b) Histograms of tomato T1 plants and control, (c) Histograms of tomato T2 plants and control, (d) Tomato T1 plants compared with control plant. (e) Chlorophyll content of mixoploid leaves from three months-old tomato T1 plants. (f) Photosynthetic rate of tomato T1 plants. Bars in C represent 50 cm. Asterisks reflect statistical significance * P ≤ 0.05, ** P ≤0.01.


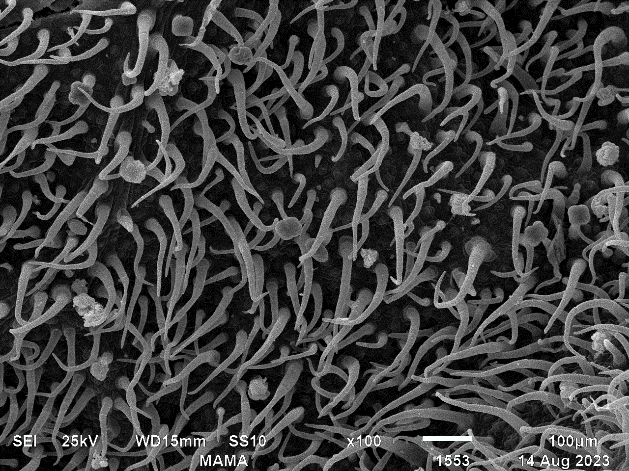

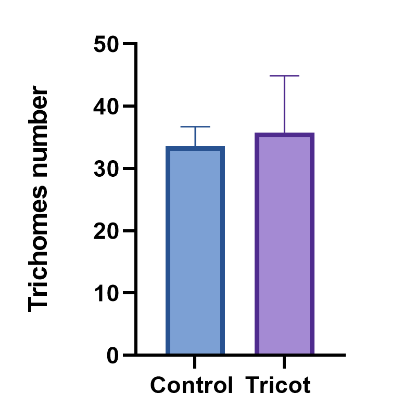

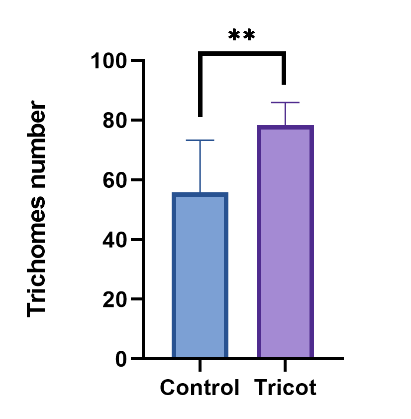


**a**

**b**

**c**

**e**

**d**


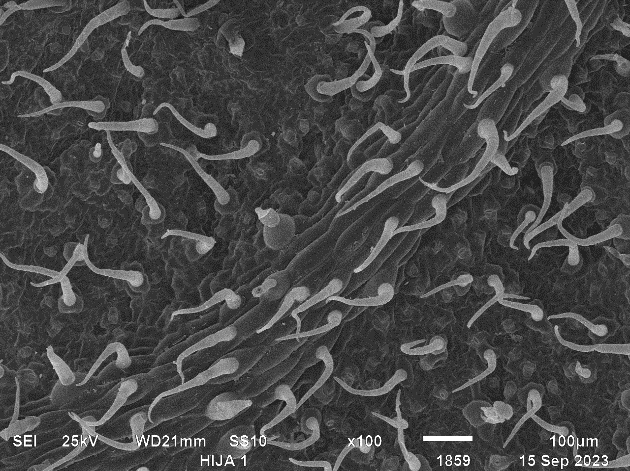

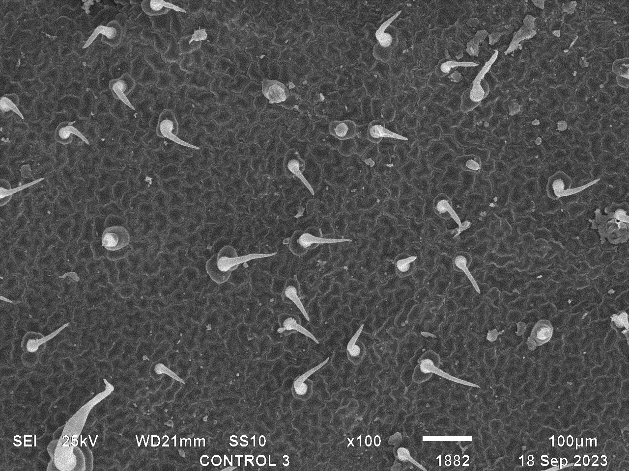

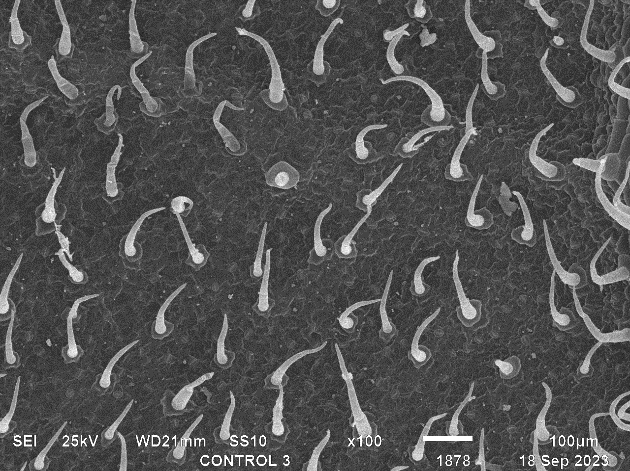

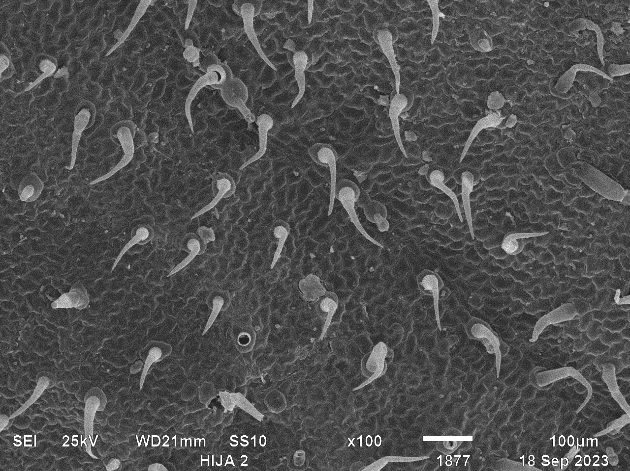


**f**

**g**

**Control**

**Control**

**Tricot N3.1**

**Tricot N3.1**


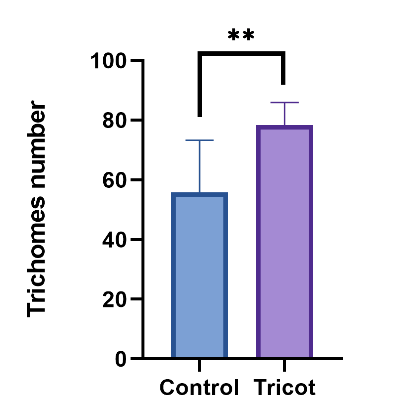

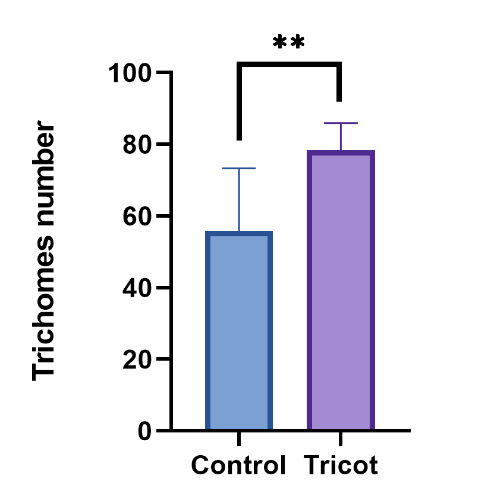

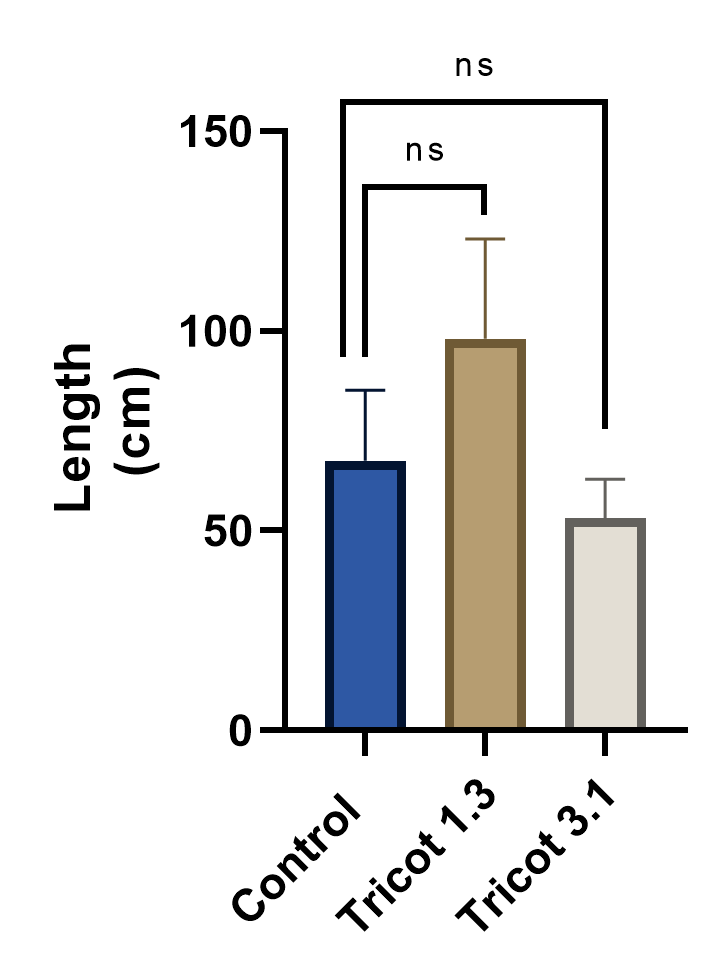


**Figure S2. Trichomes of control, T2 and T3 tomato tricot N3 plant.** (a) Scanning electron micrograph of trichomes on the adaxial leaf surface in tricot N3 plant. (b) Scanning electron micrograph of trichomes on the abaxial leaf surface in control plant. (c) Scanning electron micrograph of trichomes on the adaxial leaf surface in control plant. (d) Scanning electron micrograph of trichomes on the abaxial leaf surface in the T3 progeny of tricot N3 plant (tricot N3.1). (e) Scanning electron micrograph of trichomes on the adaxial leaf surface in the T3 progeny of tricot N3 plant (tricot N3.1). (f) Number of trichome on the adaxial leaf surface in tricot N3.1. (g) Number of trichome on the abaxial leaf surface of leaf in tricot N3.1. Asterisks reflect statistical significance ** P ≤0.01.


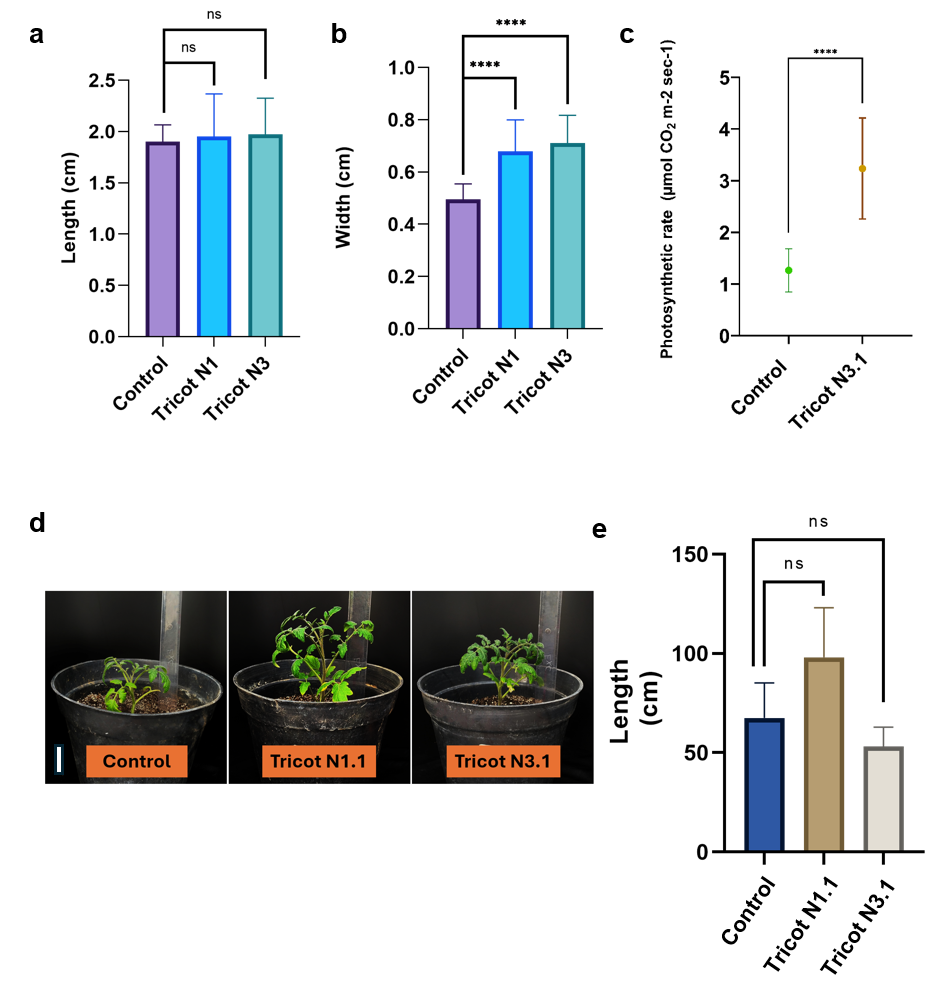


**Figure S3. Characterization of morphology T3 progeny of tricotyledonous plants. (**a) Length of cotyledons in T3 progeny of tricot N1 and tricot N3. (b) Width of cotyledons in T3 progeny of tricot N1 and tricot N3. (c) Photosynthetic rate of Tricot N3.1. (d) Three weeks-old control and tricot N1.1 ND tricot N3.1 plants (e) Length of T3 tricot N1 and tricot N3 plants after 5 months of growth. Bar in D is 5 cm. Asterisks reflect statistical significance **** P ≤ 0.0001.


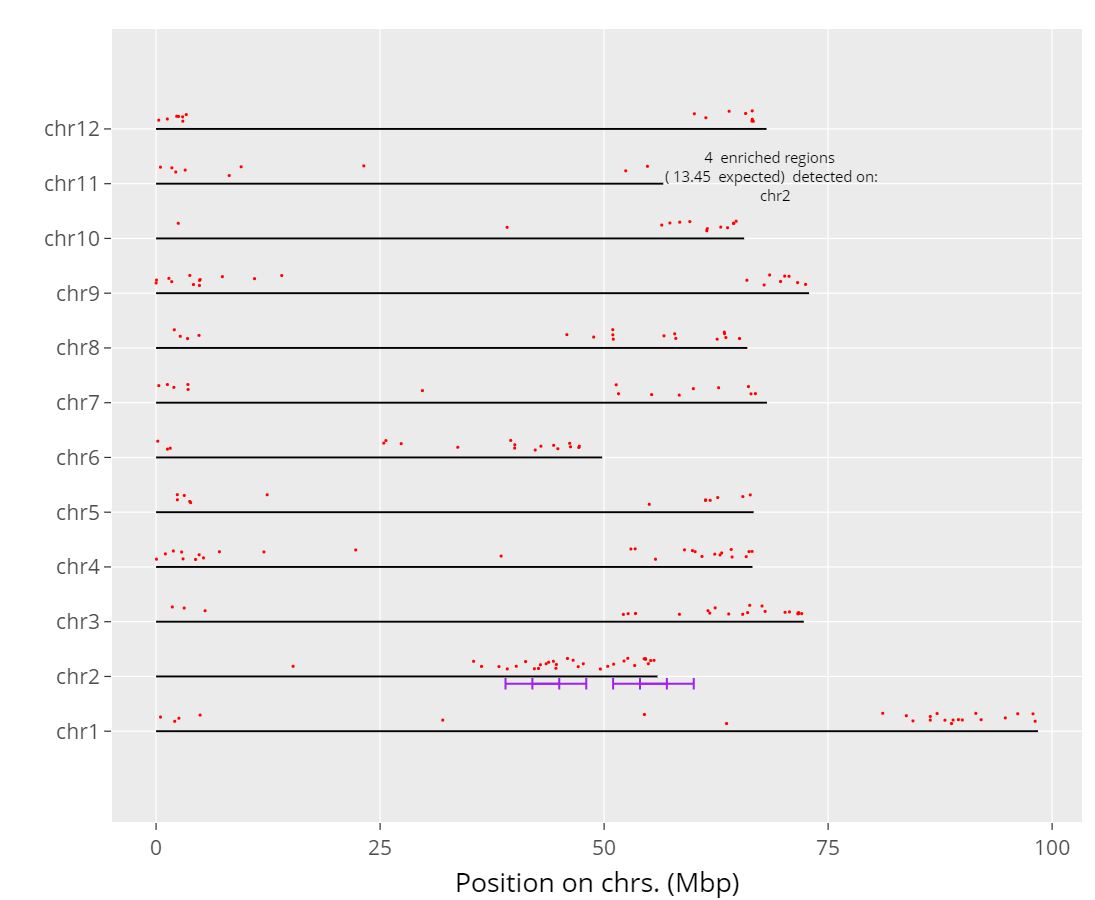

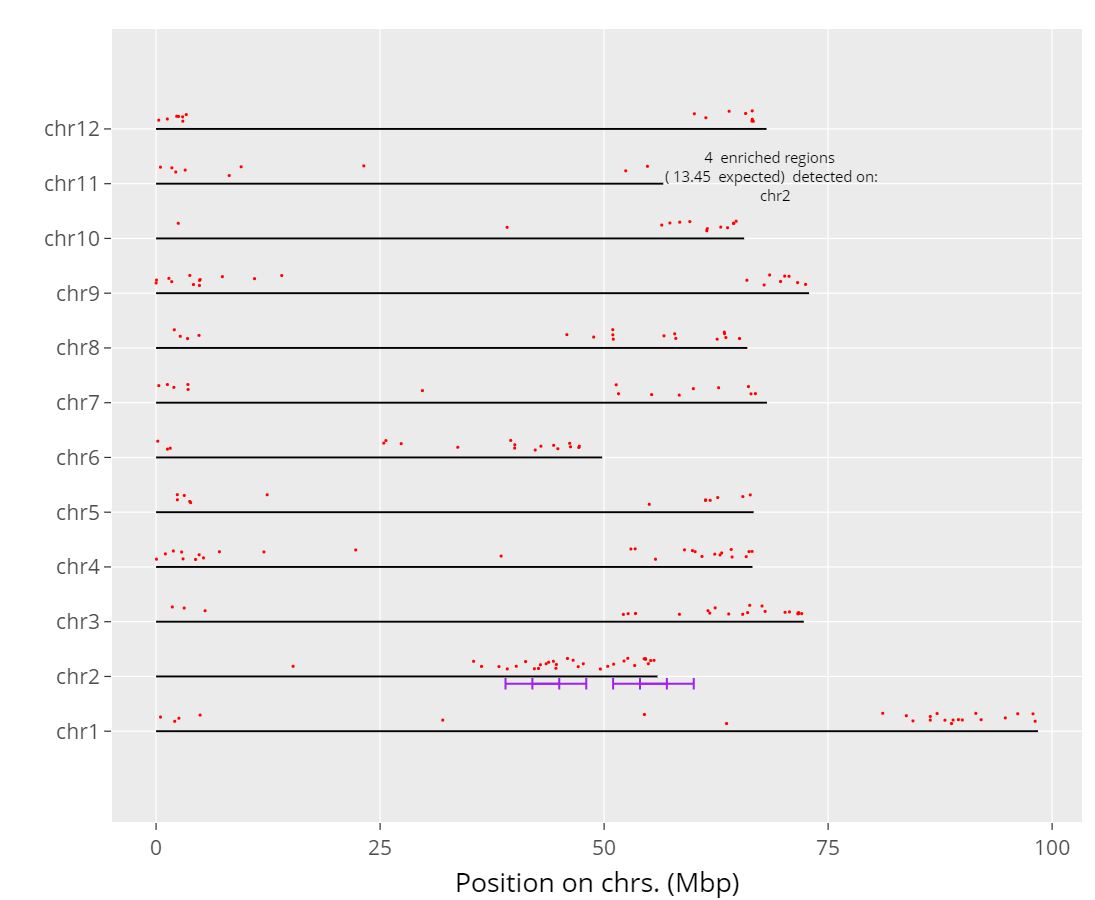

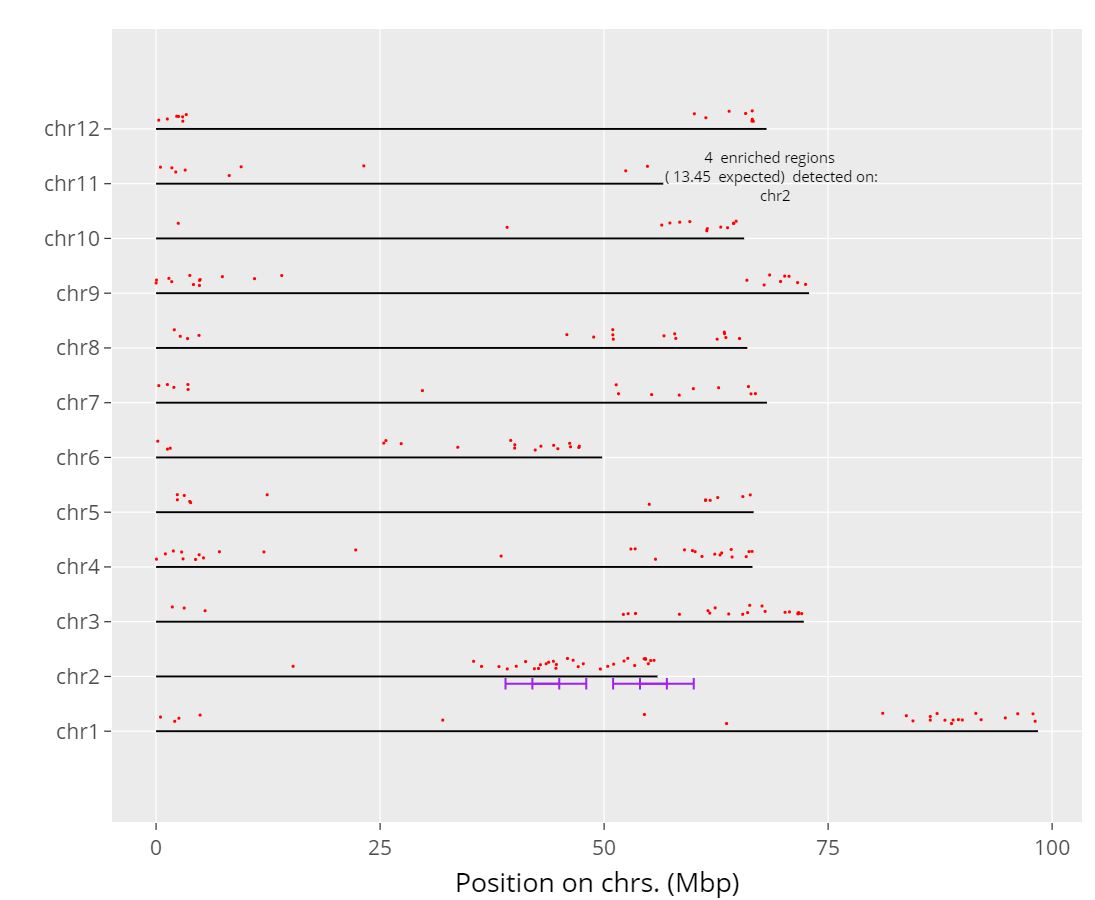


**Figure S4. Position of overexpressed genes on tomato chromosomes.** Red dots indicate genes and purple lines indicate statistically enriched regions FDR = 0.05.


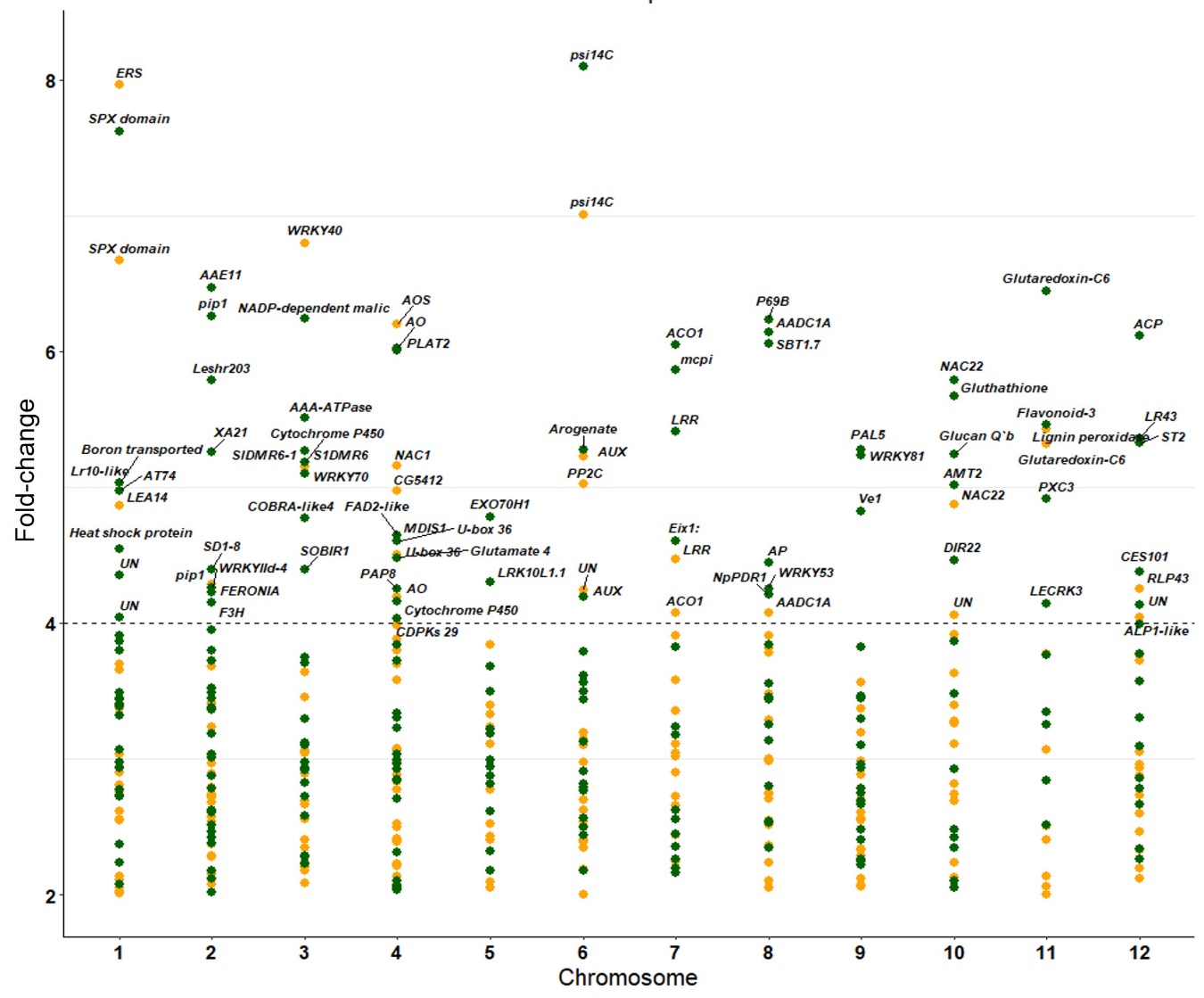


**Figure S5. Dot diagram of overexpressed genes identified on the tomato chromosomes.** The genes with the highest expression, FC > 4, stand out. Dot diagram of overlapped upregulated genes of tricot N1 and tricot N3, with the fold change of both tomato plants. Orange represents tricot N1 and green represents tricot N3.


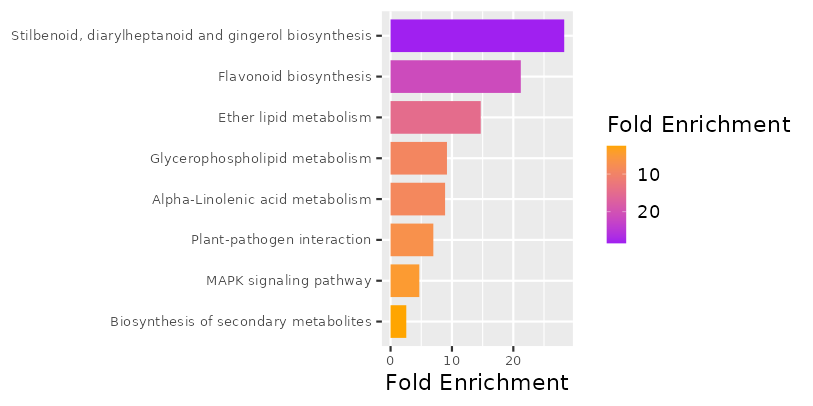


**Figure S6. KEGG enrichment of genes involved in the cotyledon ontology (PO:0020030).** Stilbenoid and flavonoid biosynthesis showed a higher fold enrichment.


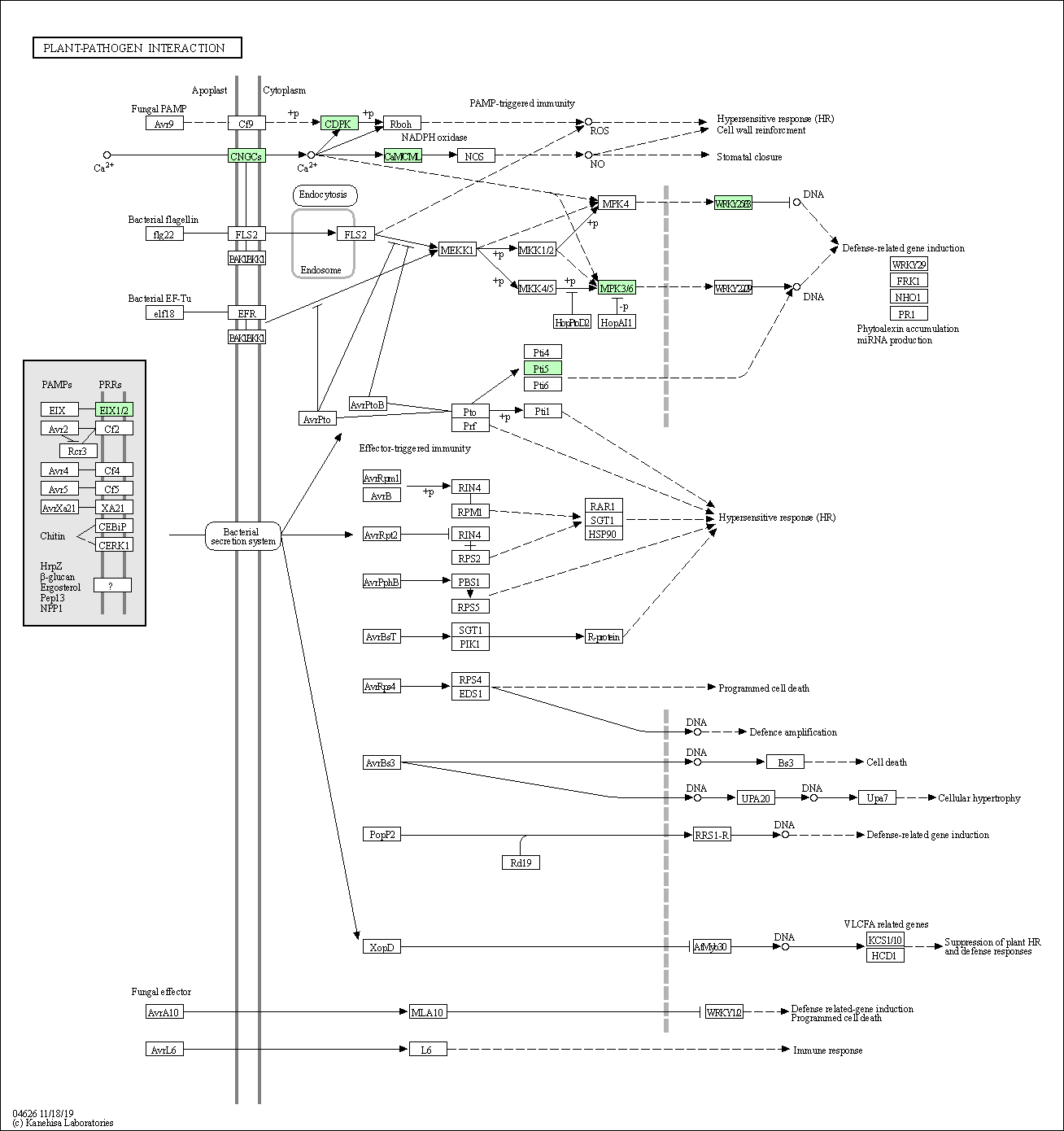


**Figure S7. Upregulated transcripts between tricot1 and tricot3 associated to plant-pathogen interactions in tricot tomato plants.** Green boxes indicate overexpression of CDPK, CNGCs, CaMCML, Pti5, MPK3, WRKY25, EIX1/2 and PR1.


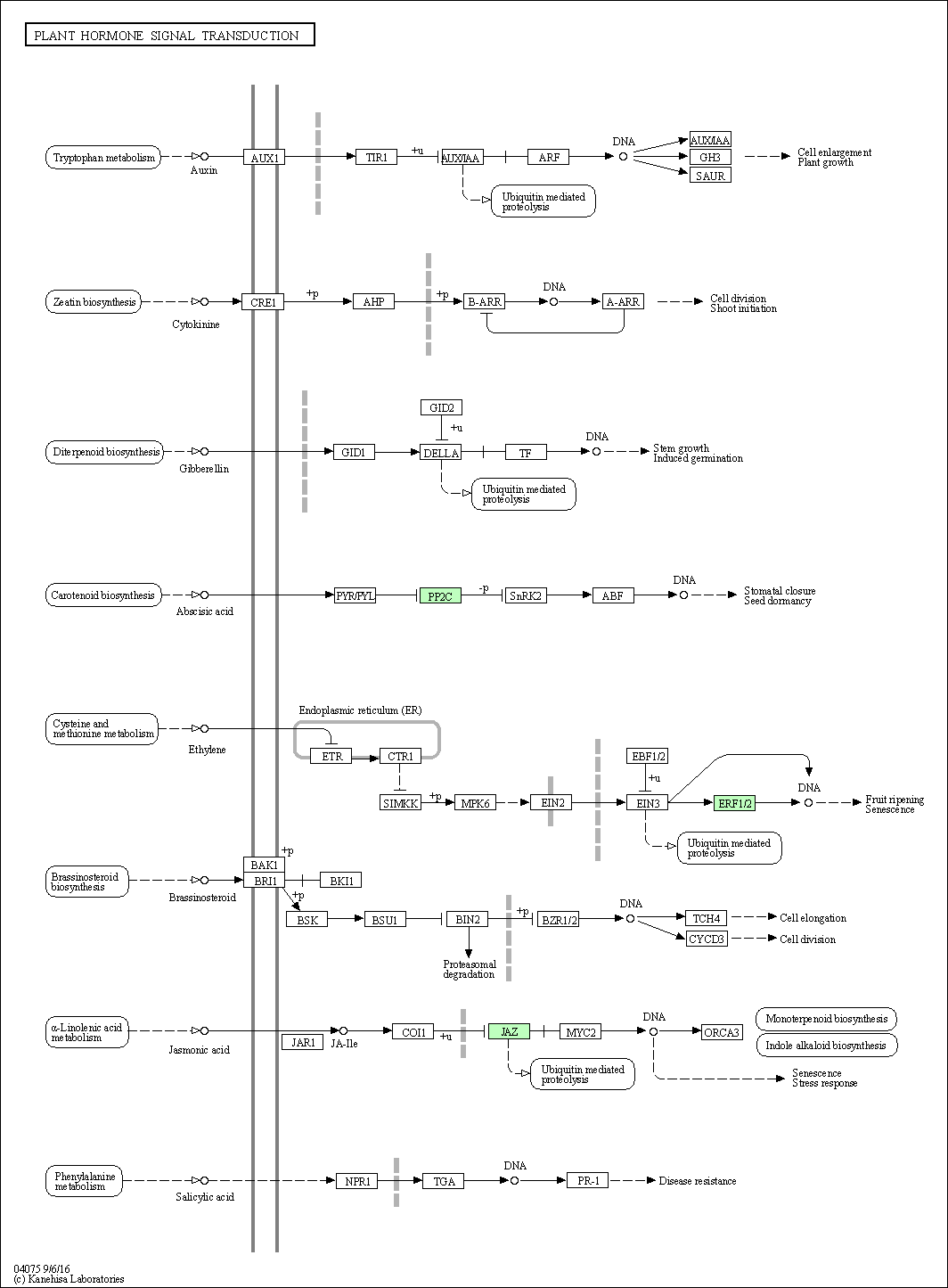


**Figure S8. Upregulated transcripts associated with plant hormone signal transduction in tricot tomato plants.** Green boxes indicate overexpression of ERF1/2, JAZ and PP2C.


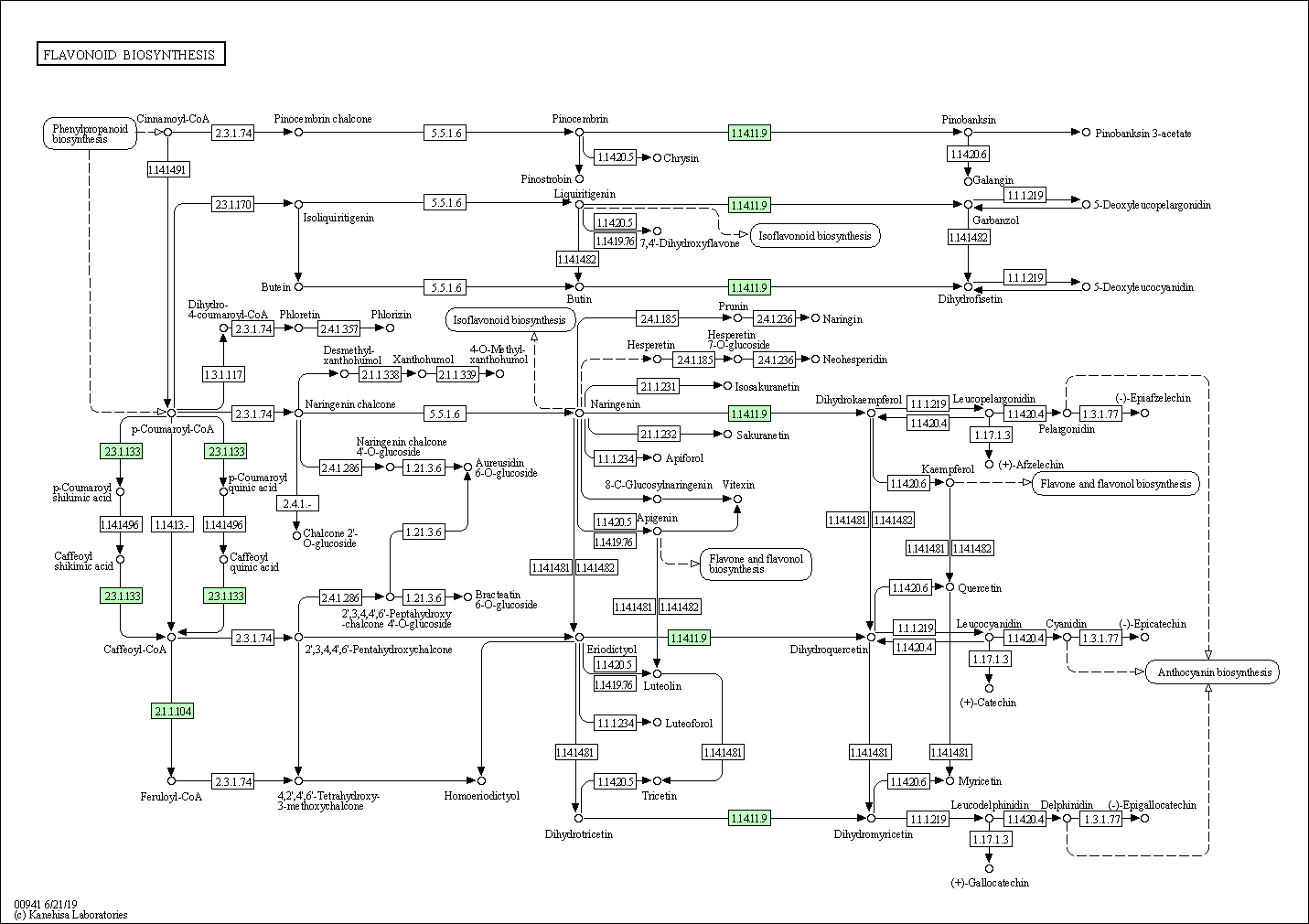


**Figure S9. Upregulated transcripts associated with flavonoid biosynthesis in tricot tomato plants**. Green boxes indicate overexpression of the genes: shikimate O-hydroxycinnamoyl transferase, caffeoyl-CoA O-methyltransferase and flavanon 3-dioxygenase.


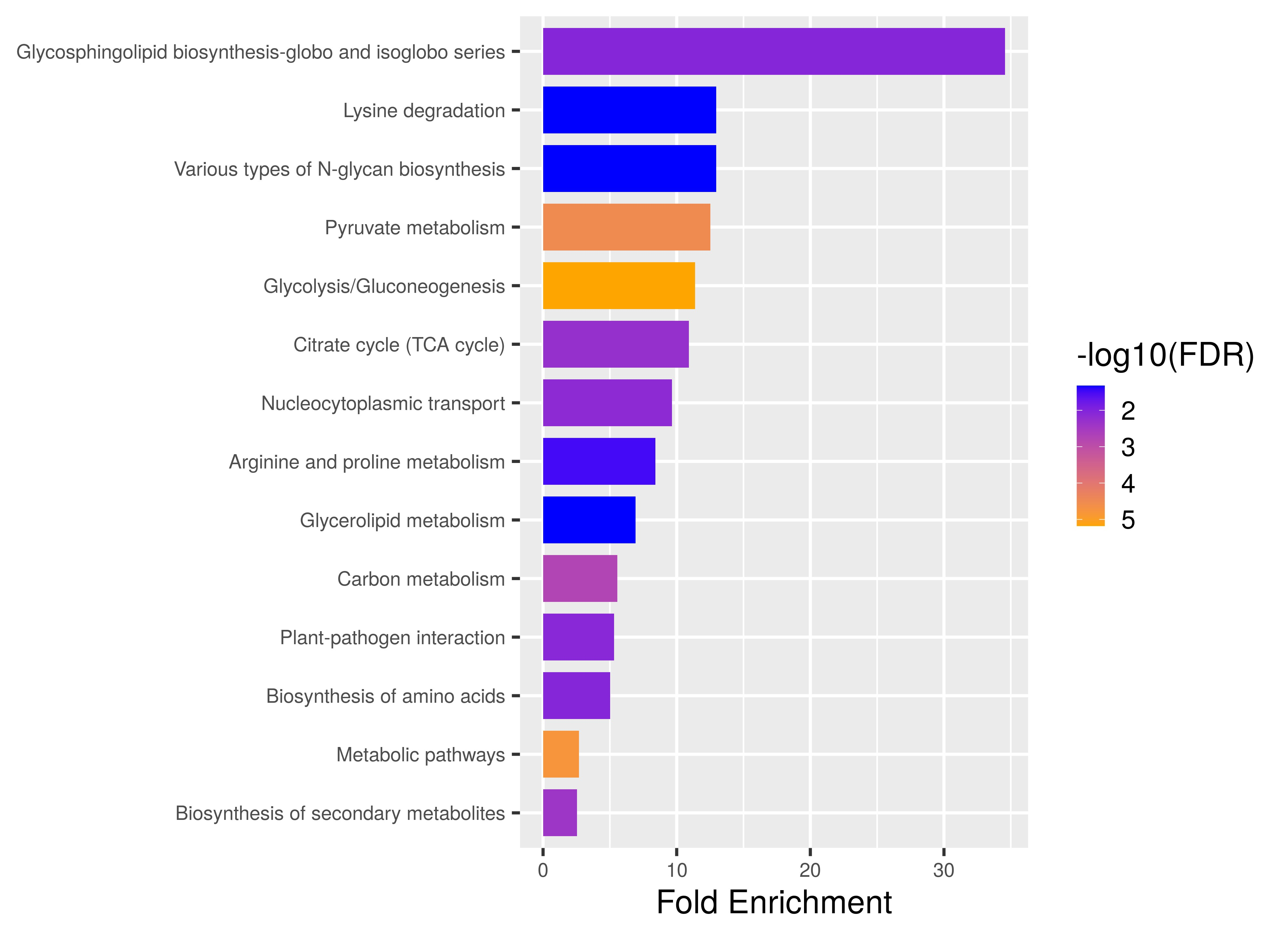


**Figure S10. KEGG enrichment of genes involved of SNP’s.** Glycosphingolipid biosynthesis-globo and isoglobo series and lysine degradation showed a higher fold enrichment.


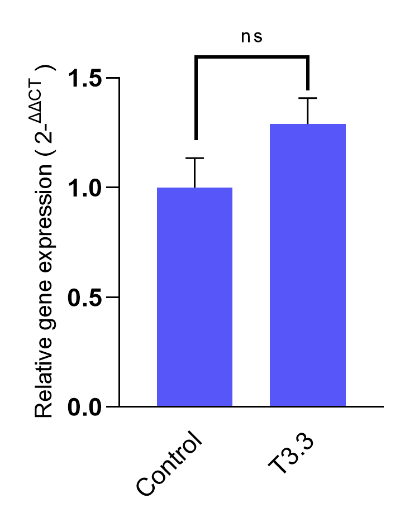

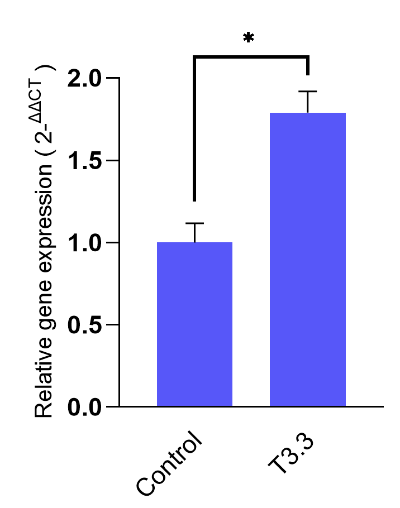

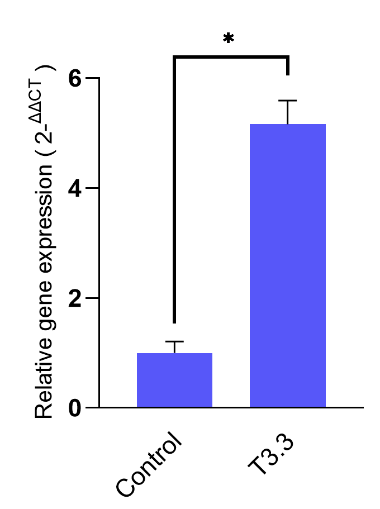

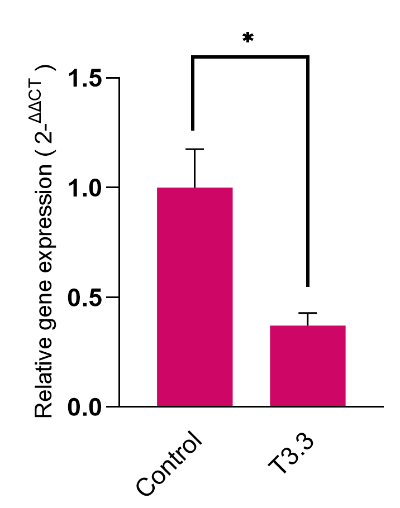

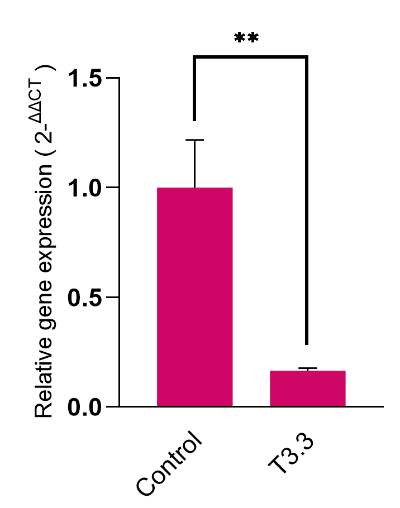

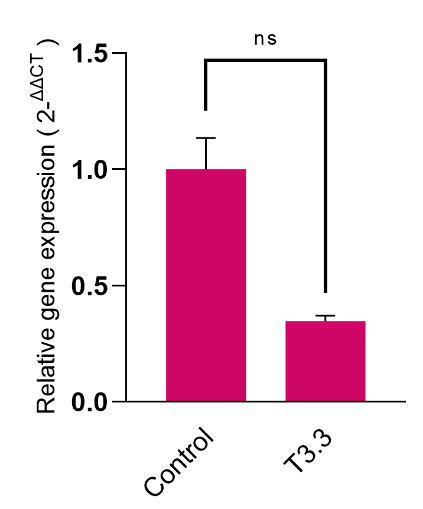


**a**

**b**

**c**

**d**

**e**

**f**

Tricot N3.1

Tricot N3.1

Tricot N3.1

Tricot N3.1

Tricot N3.1

Tricot N3.1

Control

Control

Control

Control

Control

Control

**Figure S11. Differential gene expression by RT-qPCR of T3 of N3 tricot tomato plants and control.** (a) Patatin-like protein. (b) Allene oxide synthase (aos). (c) Digalactosyldiacylglycerol synthase 2. (d) E3 ubiquitin-protein ligase RKP. (e) Putative lysine-specific demethylase JMJ16. (f) Late embryogenesis abundant protein. The graphs in blue correspond to the overexpressed genes and those in red to the suppressed genes. The bars represent the standard deviation. Asterisks reflect statistical significance * P ≤ 0.05, ** P ≤0.01.

**Table S1**. Primers used for differential expression by RT-qPCR.

| **Primer ID** | **Sequence 5´ -> 3´** |
| --- | --- |
| **XM_026028680.1_F** | GCCCAATACCAGCATCGGACA |
| **XM_026028680.1_R** | ACCAGCATCTTTGAGCATCTGGC |
| **XM_004238128.4_F** | TCTCGAATCGCAACTTCAGGAAT |
| **XM_004238128.4_R** | AGCCGTTAGCATGGCCGTTA |
| **NM_001247904.2_F** | TCGTCCCATTCAGGCTTCGG |
| **NM_001247904.2_R** | GGGCTGGGAGTTTCGCTTGT |
| **NM_001251869.2_ F** | CCAAGGGTCAAACCCAGGTGA |
| **NM_001251869.2_ R** | CTTTGGCGGAATGCGCTGTG |
| **XM_010323527.3_F** | AGAACTTGCACTGCGCAACAA |
| **XM_010323527.3_R** | GTGGCAACCCGTGTCTTAAGTG |
